# Z-DEA-FMEA: identifying effective strategies for optimizing the HIV drugs supply chain using multi-criteria decision-making approaches

**DOI:** 10.3389/fpubh.2025.1446073

**Published:** 2025-11-06

**Authors:** Amirkeyvan Ghazvinian, Bo Feng, Junwen Feng

**Affiliations:** 1 School of Economics and Management, Nanjing University of Science and Technology, Nanjing, China; 2 School of Intellectual Property, Nanjing University of Science and Technology, Nanjing, China

**Keywords:** HIV drugs, supply chain optimization, multi-criteria decision-making, Z-number, FMEA, Z-DEA, global health logistics

## Abstract

**Introduction:**

Millions of people living with HIV around the world depend on having access to antiretroviral (ARV) drugs, yet the supply chain continues to confront obstacles like rising freight costs and delivery delays. These inefficiencies put timely access to life-saving medications at risk, especially in resource-limited settings. To find ways to improve the HIV drug supply chain, this study looks into the underlying causes of these disruptions.

**Objectives:**

This study aims to: (1) assess and prioritize risks in the HIV drug supply chain, focusing on failure modes impacting delivery timelines and freight costs; and (2) enhance supply chain substantivity (fulfillment capacity) and resilience (disruption adaptability) through evidence-based strategies.

**Methods:**

Using Z-numbers to handle uncertainty, we developed a hybrid multi-criteria decision-making framework that integrates Z-SWARA, Z-WASPAS, and Z-DEA-FMEA. Along with using FMEA to assess risks and identify failure modes, the method ranks them based on freight costs and delivery timeliness, using hybrid rankings, RPN, Z-SWARA/Z-WASPAS, and Z-DEA-FMEA efficiencies.

**Results:**

Hybrid rankings indicate that the primary contributors to supply chain inefficiencies are Quantity Errors (F14, ranked 1st, 𝑄𝑡𝑜𝑡𝑎𝑙=0.9374), Pack Price Discrepancies (F16, ranked 2nd, 0.8430), and Unit Miscalculation (F13, ranked 3rd, 0.7261). The Z-WASPAS analysis emphasizes the financial implications of F16, placing it at the top for Freight Costs (*K* = 0.178). Additionally, Z-DEA-FMEA notes efficiency shifts including Delivery Confirmation (F06, 𝜃=0.7303, Delivery). In the case of Weight Failures (F20), the Freight score (𝑄𝑖=0.6991, ranked 3rd) surpasses that of Delivery (0.6753, ranked 4th), while Shipment Mode Selection (F04) holds the 5th position overall (𝑄𝑡𝑜𝑡𝑎𝑙=0.6741).

**Discussion:**

Aiming to improve the availability of antiretroviral (ARV) medications, our approach integrates risk, uncertainty, and efficiency analysis to formulate evidence-based strategies by utilizing Z-numbers. It redefines concepts of resilience and substantivity, providing decision-makers with a framework to enhance delivery speed and minimize costs. These improvements strengthen global health logistics.

## 1 Introduction

The intricate web of entities and processes comprising the supply chain for antiretroviral (ARV) treatments and HIV laboratory resources strives to ensure the timely and cost-effective provision of indispensable medicines to beneficiary nations (1–4). The World Health Organization (WHO) data underscores the gravity of the situation, revealing that 3.8 million out of the approximately 38.4 million HIV-afflicted individuals worldwide are located in Southeast Asia. This region has the unfortunate distinction of having the second-most pronounced HIV incidence, following only sub-Saharan Africa (5). Nepal, a South Asian country, has witnessed an alarming surge in HIV infection rates since the identification of its inaugural case in 1988 (6, 7). Present statistics suggest that 29,503 individuals are living with HIV in the country, translating to an HIV prevalence rate of 0.13% among adults aged 15–49. Despite rigorous endeavors to combat HIV/AIDS, the ARV supply chain faces substantial challenges, particularly in the timely and consistent delivery of drugs and laboratory resources to underserved regions (8–10). Logistical problems such as inadequate deliveries, limited infrastructure, and difficulties in reaching isolated locales remain significant hurdles (11–13). For instance, in some countries, stock shortages have led to treatment deferments, threatening health outcomes and increasing the risk of drug resistance. However, many countries are making efforts to overcome these hurdles. For example, since 2013, Tanzania has tried improving its health supply chain with an electronic logistics system. Yet, data use remained low. To fix this, the government is encouraging better use of data for smarter decisions and ongoing improvements (14).

A pivotal issue is striking a harmony between substantivity, the supply chain’s capacity to fulfill the recipient countries’ needs and resilience, indicating its robustness against disruptions and its adaptability to evolving scenarios (15).

The task of achieving this equilibrium demands meticulous considerations, including parameters like the target nation, the overseeing entity, the method of fulfillment, and the vendor’s INCO terms (16–19). Not only does HIV AIDS affect individual health, but it has deeper challenges such as economic insecurity, and also puts strains on healthcare systems especially on the supply chain management of antiretroviral drugs. Therefore, there has been great emphasis on international focusing on supply chain adaptation and treatment retention through planning and integration with the health systems (20, 21). The challenges plaguing the ARV and HIV lab supply chain, such as stock shortages, treatment deferments, and the looming threat of drug resistance, remain pressing concerns that require urgent attention (22). These challenges exacerbate the health risks for those living with HIV and catalyze the further transmission of the virus. Optimization of this supply chain is non-negotiable in the quest to terminate the HIV epidemic (13, 23). Central to this mission are data-centric methodologies that refine the supply chain’s efficacy. The focus of this study is the exploration of the equilibrium between substantivity and resilience in the ARV and HIV lab supply chain directed toward the assisted nations (24, 25). This comprehensive approach facilitates an exhaustive examination of diverse supply chain-associated variables. Gleaned insights from these analytical procedures are poised to arm stakeholders and policy architects with invaluable data, fostering more informed decision-making. It is anticipated that this will enhance the supply chain performance, strengthening the global resistance to HIV/AIDS. By pinpointing and emphasizing critical factors like the destination country, managing entity, fulfillment modality, and vendor INCO specifications, the study seeks to pave the way for supply chain enhancements that promise improved health outcomes for those with HIV. Further, optimizing this supply chain can precipitate cost reductions in treatment and broaden the accessibility of antiretroviral medications and diagnostic tools (26). In essence, the insights and recommendations proffered in this research endeavor aim to be a cornerstone in the collective global ambition of eradicating the HIV epidemic, by augmenting the competency and impact of the HIV drug supply chain to nations in need.

Effective distribution and utilization of ARVs depend on hospital efficiency, as a key endpoint in the supply chain. Data Envelopment Analysis (DEA) has been used extensively to measure hospital performance and provide insights that can be applied to supply chain optimization. For instance, Lindlbauer et al. (27) evaluated German hospitals after quality certification, Li and Dong (28) used bootstrap-DEA for Chinese public hospitals, and O’Neill et al. (29) provided a cross-national DEA taxonomy for hospital efficiency. Recent studies, such as Chowdhury et al. (30) on Indian private hospitals and Li et al. (31) on physician-hospital integration, have highlighted the role of DEA in identifying inefficiencies under resource constraints, directly informing ARV supply strategies in resource-limited settings. Additionally, Nunes and Ferreira (32) used network DEA to assess the efficacy and efficiency of Portuguese public hospitals before and after COVID-19, observing initial declines but recoveries with improved patient safety—demonstrating the usefulness of DEA for resilience in interrupted systems. Similar to our focus on freight optimization in ARV supply chains, Ferreira et al. (33) created a log-linear DEA model for pay-for-performance in hospitals, delivering cost reductions by matching compensation with quality and access. Our Z-DEA-FMEA integration is informed by these studies, which highlight DEA’s flexibility in the face of uncertainty.

Our research integrates Z-SWARA, Z-WASPAS, and Z-DEA-FMEA multi-criteria decision-making (MCDM) techniques with the Failure Modes and Effects Analysis (FMEA) method, forming a hybrid framework to identify effective strategies for optimizing the HIV drug supply chain, with a focus on delivery scheduling. This data-centric approach enhances supply chain performance by addressing inefficiencies through a structured methodology, chosen for the following reasons:

Z-SWARA, Z-WASPAS, and Z-DEA-FMEA: These approaches help ranking and prioritization of supply chain factors. They integrate risk and efficiency, using Z-numbers to address uncertainty in Severity, Occurrence, and Detection assessments and also, they facilitate structured decision-making based on identified factors for both delivery and freight efficiency.

FMEA Method: Failure Modes and Effects Analysis (FMEA) systematically identifies risks (e.g., quantity errors, weight failures) impacting freight costs and delivery timelines. This supports proactive mitigation of supply chain vulnerabilities, such as stock shortages.

Z-DEA-FMEA and Hybrids: Z-DEA-FMEA evaluates supply chain operations’ efficiency and offers information about performance gaps to guide optimization.

The steps of the proposed method are as follows:

1 Collect data on ARV and HIV lab shipments and assess risks using FMEA (Severity, Occurrence, Detection).
2 Define failure modes and assess risks using expert opinions and FMEA outcomes.
3 Develop a multi-criteria decision-making model integrating Z-SWARA, Z-WASPAS, and Z-DEA-FMEA, using Z-numbers for optimizing delivery timelines and freight costs, enhancing substantivity and resilience.
4 Rank supply chain factors using RPN and Z-SWARA/Z-WASPAS to prioritize risks.
5 Assess failure mode substantivity and resilience using Z-DEA-FMEA efficiencies to guide prioritization.
6 Compute hybrid rankings and combined rankings (see Results section).

The structure of this paper is organized as follows: We begin with an introduction, followed by a related work review, methodology (data collection, proposed approach), results, discussion, conclusion, and future research directions.

## 2 Related works

Medical male circumcision and pre-exposure prophylaxis (PrEP) have been shown to reduce the burden of HIV among men (34–36). Naidoo et al. (21) and Cremin et al. (37) investigated ART and PrEP coverage rates in KwaZulu-Natal, South Africa. Identifying HIV-positive individuals one-year post-infection and elevating ART coverage to 80% could potentially avert 35% of cumulative HIV infections across a decade. However, the impact may be attenuated due to the surge in newly infected individuals. Targeted PrEP, when given to couples at the onset of infection, yields an incremental cost-effectiveness ratio of $40,000 per averted infection. In a related study, Alsallaq et al. (38) in KwaZulu-Natal, South Africa, modeled similar interventions to discern if they exhibited synergistic outcomes. Their findings suggest that the most pronounced synergistic effects arise when interventions are compromised by factors like behavioral disinhibition, suboptimal adherence, and ART discontinuation. In the broader spectrum of HIV prevention, strategies encompassing both medical male circumcision and PrEP could be pivotal if they further curtail transmission rates. However, to offer ART to the global count of 34 million HIV-positive individuals, a staggering $19.1 billion in international funds remains a requisite (39). While it is crucial to augment ART coverage, several challenges and considerations remain at the forefront. Aspects tied to each stage of the models mentioned above, such as testing frequency, care uptake ratios, adherence, and attrition rates, warrant meticulous scrutiny. To ensure sustained viral suppression, patients need to navigate a care cascade, delineating their treatment initiation, ART commencement, subsequent follow-ups, and the achievement of consistent viral suppression. Furthermore, dynamic models serve as invaluable tools for operational research. They can spotlight logistical challenges and factors hampering health outcomes and guide the judicious allocation of resources, grounded in defined objective functions (40).

While taking into account how the entire supply chain influences costs is important, it is crucial to note that this perspective may not fully encapsulate health outcomes. Brown et al. (41) modeled the vaccination supply chain in Benin using a discrete-event, agent-based framework. Their study examined a variety of scenarios, such as streamlining administrative tiers, transitioning to bikes for direct deliveries, and utilizing trucks for looped deliveries. Particularly noteworthy was the shift from existing “Health Zones” to third-tier “Communities” and the replacement of truck loops with motorcycle deliveries; these changes collectively reduced the logistics cost per vial from $0.26 to $0.19 in 2017, a total savings of $500,000. Such enhancements might be advantageous for HIV initiatives.

Assi et al. (42) investigated the efficacy of vaccination supply chains in Niger by simplifying the number of tiers. By excluding regional-level outlets and allowing district stores to directly fetch vaccines from central repositories, vaccine availability surged to 95%, and the cost per dose slightly dropped. Past studies, like those involving One Health (43) and Supply Chain Guru (44), have probed into how supply chains impact health. In earlier disease models, there is a direct correlation between supply networks and health outcomes. It is critical to precisely project ART availability, ART dispatch, and health outcomes within HIV care cascades at clinic tiers (45, 46). Mathematical modeling offers a lens to further dissect the HIV supply chain. For instance, similar to pharmaceuticals, laboratory tests are location-bound. Past spatial analyses, as seen with polio in Nigeria (47) and HIV in Kenya (48), can be instrumental in optimizing laboratory test deliveries. Moreover, to cater to the 34 million HIV patients, manufacturing processes demand recalibration, ensuring production amplification dovetails with reduced acquisition costs (49).

Accurate forecasting models are indispensable to temper financial unpredictability and smoothen pharmaceutical production processes. Despite its popularity, the HIV rapid test supply chain is restricted, making its management arduous, particularly under constrained resources. The diagnostic market grapples with supply inconsistency, convoluted procurement, delays, and inadequate quality assurance. Compromised supply chains could potentially deteriorate point-of-care testing outcomes (2). Biza et al. noted that flawed supply chains stymied the distribution of prenatal care kits across three health institutions in Mozambique (50). To adeptly infuse POC technologies, procurement frameworks equipped for regular cold chain management and limited shelf-life are pivotal (1–3, 51). There are a number of roadblocks to the effective implementation of Point-of-Care (POC) diagnostic systems, especially the lack of resources and inadequate handling of inventory management systems as well as problems relating to congestion of the Health Facility. Such bottlenecks can delay the distribution process, most especially in resource actualized situations where logistical obstacles and slow mechanisms of requesting for tests restrict quick diagnosis (1, 3, 52). Ensuring quality in LMICs is further complicated by gaps in knowledge and training (53, 54). Velloza et al. note that HIV prevention strategies must be community-specific. The investigations highlight that adequate service delivery requires knowledge of specific characteristics of the area variations, including local culture and health system components. This includes tackling supply chain and human resources challenges in order to coordinate with local authorities for better implementation of the strategies. In this respect, they stress that factors related to context should not be downplayed because they could alter the acceptance and the quality of the HIV prevention products, thus it is critical to integrate the views of the community in the decision-making processes (55). Additionally, Engel et al. observed a deficit in user representation during R and D, leading to POC diagnostics that might be misaligned with their intended use-cases. Addressing these challenges requires an emphasis on localized and transdisciplinary operational research for the POC diagnostics value chain system (56, 57). Yazdanparast et al. (58) introduced a hybrid method combining Z-number Data Envelopment Analysis (Z-DEA) with neural networks to assess supply chain resilience. This approach leverages expert insights to evaluate 16 resilience enablers within an Iranian automotive supply chain. Z-DEA incorporates uncertainty and reliability into the analysis, while neural networks handle complex, non-linear relationships, demonstrating that targeted improvements in resilience enablers can mitigate supply chain disruptions. Karuppiah et al. elaborations on supply chain management and sustainability offers valuable insights for optimizing systems, providing frameworks for enhancing resilience and efficiency that could inform our HIV supply chain strategies (59, 60).

Khadem et al. (61) investigated the role of Artificial Intelligence (AI) in optimizing supply chain operations, emphasizing applications like demand forecasting, inventory management, and transportation. Although the study does not directly address Z-numbers, it highlights how advanced computational tools can enhance efficiency, reduce costs, and improve decision-making, aligning with the objectives of Z-number methodologies for managing uncertainty and ensuring reliability.

Muysinaliyev and Aktamov (62) provided a broad review of supply chain management, covering key concepts, performance metrics, and challenges. While Z-numbers were not a focus, the study underscores the need for advanced tools to address evolving complexities in supply chain practices. It emphasizes the importance of integrated strategies to boost performance, aligning with the goals of Z-number-based approaches for addressing modern supply chain challenges.

Recent advancements in multi-criteria decision-making, including Z-SWARA and Z-WASPAS, enhance FMEA with fuzzy Z-numbers, while Data Envelopment Analysis (DEA) measures efficiency. However, integrating these into hybrids like Z-SWARA-DEA-FMEA and Z-WASPAS-DEA-FMEA remains underexplored. Tables 1, 2, provide a summary of related methods and contextual literature on multi-criteria decision-making in healthcare and supply chain and an overview of challenges and prospects in the HIV supply chain, respectively.

**Table 1:** Review of related methods and contextual literature on multi-criteria decision-making in healthcare and supply chain.

Author	Year	Method	Result
Oluwadare et al. (81)	2024	integrates fuzzy VIKOR and entropy methods	The study introduces a framework that integrates fuzzy VIKOR and Entropy methods to assess Healthcare 4.0 technologies, emphasizing crucial technical and economic aspects of supply chain management. It provides recommendations for technology adoption in developing countries, based on insights from data gathered in Lagos, Nigeria.
Sawik et al. (82)	2023	Multi-objective optimization	A multi-criteria optimization model and a hands-on approach illustrate the interaction between risk, sustainability, and supply chain with space mission planning, organization, and execution.
Karbassi Yazdi et al. (83)	2022	Hesitant fuzzy sets (HFS)—MABAC	An effective method for computing resilience-related CSFs in choosing transportation service providers under ambiguous conditions can be derived from analyzing a hybrid MCDA approach.
Shakibaei et al. (84)	2024	MCDM	This study aims to explore humanitarian supply chain models by developing a new framework that optimally establishes essential relationships to reduce human, financial, and moral losses. The proposed model demonstrates high accuracy based on algorithm design, problem modeling, and case study findings.
Boonsothonsatit et al. (85)	2024	TOPSIS and AHP	The study highlights that the top three prioritized criteria across hospitals are budget and funding support (BF) (0.329), staff proficiency with technology (TP) (0.147), and leadership support (LS) (0.128). These findings provide valuable guidance for healthcare organizations in making informed technology implementation decisions, especially to improve medication dispensing processes.
Abhilasha et al. (86)	2024	Fuzzy VIKOR	This study proposes a two-phase approach using fuzzy VIKOR to rank suppliers and MOLP to minimize costs, delays, and emissions. A case study in a pharmaceutical company identified maritime transport as the most eco-friendly option compared to air and truck transport.
Senapati et al. (87)	2024	Fuzzy-based Decision-Making with Sugeno-Weber Operations	Sugeno-Weber t-conorms and t-norms in a Dual Hesitant q-Rung Orthopair Fuzzy (DHq-ROF) were used to improve the precision, decrease uncertainty, improve resource allocation and enhance decision-making in medical supply chains.
Moosivand et al. (88)	2021	AHP and TOPSIS	Drug shortage management strategies were prioritized using TOPSIS for ranking and AHP for weighting criteria. Highlighted how supply chain management, policymaking, and information systems may improve medicine availability.
Gumede et al. (21)	2022	Descriptive analysis	Investigated ART and PrEP coverage in KwaZulu-Natal, South Africa. Findings indicate that targeted PrEP for couples at the onset of infection yields an incremental cost-effectiveness ratio of $40,000 per averted infection.
Alsallaq et al. (38)	2023	Dynamic modeling	Showed synergistic outcomes in HIV prevention strategies but emphasized challenges such as behavioral disinhibition and suboptimal adherence.
Brown et al. (41)	2017	Agent-based framework	Modeled vaccination supply chains in Benin, highlighting cost reductions by streamlining delivery methods and transitioning to direct and looped deliveries using motorcycles.
Assi et al. (42)	2017	Simplified tiers in supply chains	Simplified vaccination supply chains in Niger by reducing tiers, leading to a 95% vaccine availability rate and lower costs.
Engel et al. (57)	2020	Localized operational research	Identified the need for better user representation in R&D and emphasized community-specific approaches for HIV Point-of-Care (POC) diagnostics.
Velloza et al. (55)	2021	Community-specific approaches	Highlighted the importance of tailoring HIV prevention strategies to local contexts, including supply chain and human resource integration for effective implementation.
Damtie et al. (25)	2020	Supply chain management performance of HIV/AIDS	Only 30% of health facilities received ARV drug orders on time, with average lead times of 46.4 days in hospitals and 59.2 days in health centers. Frequent stockouts, emergency orders, and inventory inaccuracies were reported, although 83.3% of facilities maintained adequate storage conditions.
Berhanemeskel et al. (22)		HIV/AIDS related commodities supply chain management	The study found that most health facilities faced frequent stockouts and emergency orders for ARV medicines and test kits, with nearly 75% experiencing shortages during the study. Despite some timely reporting, gaps in service delivery and supply management were evident across hospitals and health centers.
Stulens et al. (89)		introduction to HIV supply chains in low- and middle-income countries	The authors conclude the study by highlighting key trends, identifying existing gaps, and suggesting future directions for research in Operations Research (OR) and Operations Management (OM)

**Table 2:** Overview of challenges and prospects in the HIV supply chain.

Challenges	Responses by global HIV initiatives	Opportunities for NCD supply chain	Opportunities
Unreliable funding sources	Support for domestic fund gathering Aid for global fund collection	Further enhancement in gathering domestic resources Persist in effectively vying for international funding opportunities	Which innovative financing approach is the most effective for supply chain fund mobilization?
Insufficient workforce availability	Cultivation of local supply chain teams Temporary technical support and a more intelligent, strategic method for nurturing and retaining supply chain personnel	Enhance training collaborations and syllabuses Skills adaptable for NCD supply chain utilization	Which innovative methods in hiring, training, and employment can address the deficits in the supply chain workforce?
Inadequate infrastructure and tangible assets Efficient use of resources	Enhanced regional distribution facilities Optimized storage solutions Cost-efficient, high-value strategies for warehouse layout	Utilize and broaden current infrastructure enhancements, encompassing storage, transport, and IT resources	What are the cutting-edge, economical designs for storage and transportation specifically tailored for housing and dispersing NCD goods?
Flawed purchasing systems	Negotiated product costs Consolidated acquisitions Strategic supply forecasting Encouraging global benchmarks with regional suppliers	Harness and proliferate insights acquired to enhance NCD procurement techniques	Which benchmarks and tech solutions should be universally adopted to guarantee superior NCD service and product distribution?

## 3 Methodology

### 3.1 Dataset

The Global Fund and PEPFAR, major procurers of HIV health commodities, rely on the Price, Quality, and Reporting (PQR) dataset. Comprising 10,325 shipment records, this dataset provides comprehensive insights into global HIV expenditure, price ranges, shipment trends, and country-specific volumes. Supplemented by expert opinions, it reliably informs analyses of weight, quantity, and delivery timelines despite limitations in real-time data. Stakeholders can leverage this resource for informed decision-making, as endorsed by the USAID (63), to enhance HIV drug logistics.

### 3.2 Triangular fuzzy numbers in Z-number framework

Fuzzy set theory was developed by Zadeh (64). A fuzzy set theory extends classical set theory to solve practical problems in uncertain environments. A fuzzy set $\tilde{a}$ is a pair (U, m), where U is a set and m: *U → [0, 1]* is a $\mu_{\tilde{a}}$ (*x*) denoted membership function mapping elements to a degree of belonging between 0 and 1.

Definition 1. Let $\tilde{a}\in$ *F* (*R*) be a fuzzy number if:

(A) There is *x* 0 ∈ *R* such that $\mu_{\tilde{a}}$ (x_0_) = 1;
(B) For any *α* ∈ [0, 1], $\tilde{a}$ _α_ = [x, $\mu_{\tilde{a}}$ α(*x*) ≥ α] is a closed interval. Here, the set of real numbers is R, and *F* (R) describes the fuzzy set.

Definition 2. If its membership functions $\mu_{\tilde{a}}$ (*x*): *R* → [0, 1], a Fuzzy number $\tilde{a}$ on R is known as a Triangular Fuzzy Number (TFN).

Is identical to Equation 1


$$(x)\mu_{\tilde{a}}=\{\begin{array}{c} \begin{array}{c} \begin{array}{c} 0\;\;\;x<l\\ \begin{array}{cc} \frac{x-l}{m-l} & l\leq x\leq m \end{array} \end{array}\\ \begin{array}{cc} \frac{u-x}{u-m} & m\leq x\leq u \end{array} \end{array}\\ \begin{array}{cc} 0\; & x>u \end{array} \end{array}$$ (1)

**Figure 1:**
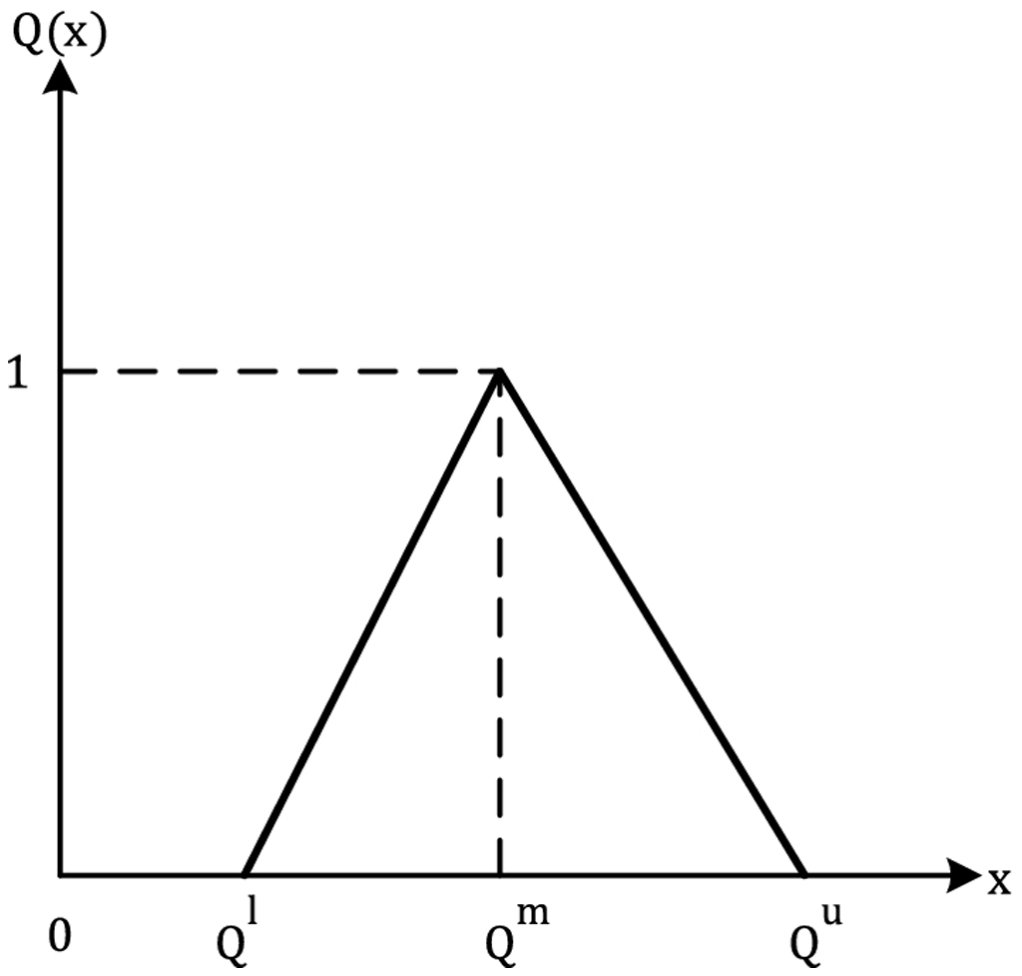
Fuzzy triangular number.

In this formula, l, m, and u are the lower, modal, and upper support values, which are all crisp (−∞ < l ≤ m ≤ u < ∞). As a triplet of letters (l, m, u), a TFN can be represented. Two TFNs are described in (65) for a basic understanding of how they work (Figure 1; Table 3).

**Table 3:** The linguistic variables that determine decision-makers’ transformation rules.

Linguistic terms	Membership function
Equally importance (EI)	(1, 1, 1)
Weakly important (WI)	(2/3, 1, 3/2)
Fairly important (FI)	(3/2, 2, 5/2)
Very important (VI)	(5/2, 3, 7/2)
Absolutely important (AI)	(7/2, 4, 9/2)

Definition 3. To convert a TFN( ${\tilde{a}}_{i}$ ) = ( $l_{i},m_{i},u_{i}$ ), to a crisp value for analysis, we use the simple average defuzzification method Equation 2:


$$R\left({\tilde{a}}_{i}\right)=\frac{l_{i}+m_{i}+u_{i}}{3}$$ (2)

This method applied to defuzzify TFNs in Z-SWARA, Z-WASPAS, and Z-DEA-FMEA calculations.

### 3.3 Z-number theory

As Zadeh (66) introduced a framework for mathematically representing linguistic claims, this kind of probability-qualified statement was introduced. A Z-number $\left(\tilde{F},\tilde{L}\right)$ is a pair of ordered numbers in which both $\tilde{F}$ and L is TFNs. Y domain is comprised of the fuzzier $\tilde{F}\;$ as an initial component. The variable Y represents an uncertain real-valued variable, while the variable Z represents its Z-number. An interval between [0, 1] is represented by the fuzzy subset $\tilde{L}$ . Restrictions are represented by $\tilde{F}$ and dependability by $\tilde{L}$ . Z-numbers can communicate unknown variables. There is a collection of Z-values that constitute Z-information. Several everyday decisions and thoughts are based on Z-information. Assume that Y, in Equation 3, is a fuzzy variable since it is a stochastic variable. A probability distribution of Y indicates the probability of Y in the Equation. The probabilistic constraint is as follows.


R(Y):Yisp (3)

Using Equation 3 as a starting point, it can be seen that the function of Y’s probability density can be found in Equation 4.


R(Y):Yisp→prob(u≤Y≤u+du)=p(u)du (4)

Y is represented by the probability density function p, and u is represented by the differential du.

Z-numbers can be converted to TFN by assuming $Z=\left[\left(a_{1},b_{1},c_{1}\right),\left(a_{2},b_{2},c_{2}\right)\right]$ . Restrictions are represented by the initial component $\left(a_{1},b_{1},c_{1}\right)$ . Reliability is represented by the second component $\left(a_{2},b_{2},c_{2}\right)$ . The second component (reliability) is converted into a crisp integer using the centroid method as follows (67):


$$\alpha =\frac{\int y\;\mu_{\tilde{L}}\left(y\right)dy}{\int \mu_{\tilde{L}}\left(y\right)dy}\;$$ (5)

$\mu_{\tilde{L}}\left(y\right)$ equals, as shown in Equation 6. Due to this, the second factor (reliability) has the same weight as the first (restriction). The weighted Z-number in TFN form can be obtained by applying:


$${\tilde{Z}}^{\prime }=\left(l_{A}\cdot \alpha ,m_{A}\cdot \alpha ,u_{A}\cdot \alpha \right)$$ (6)

For triangular fuzzy reliability components, the centroid method (5) simplifies to $\alpha =\frac{l_{B}+m_{B}+u_{B}}{3}$ .

### 3.4 Z-SWARA method

The Stepwise Weight Assessment Ratio Analysis (SWARA) method, introduced by Kersuliene et al. (68), determines criteria weights through expert judgments in a structured manner. Fuzzy SWARA extends this by using Triangular Fuzzy Numbers (TFNs) to handle uncertainty in expert opinions. In this study, we further extend fuzzy SWARA to Z-SWARA by incorporating Z-numbers, which add reliability to the weight assessment process, enhancing the credibility of the results (69). Experts compare criteria step-by-step, assigning relative importance based on the cumulative influence of preceding criteria. The resulting weights (Table 4) are used in Z-WASPAS (Table 5) and hybrid rankings (Tables 6–8). The Z-SWARA method involves the following steps:

**Table 4:** Z-SWARA criteria weights.

Objective	Criterion	Z-number	K_j_	Final weight (w_j_, TFN)
Delivery to Client	S	[(2.5, 3, 3.5), (0.75, 0.9, 1)]	–	(0.47, 0.54, 0.62)
	O	[(1.5, 2, 2.5), (0.65, 0.8, 0.95)]	0.4	(0.33, 0.36, 0.39)
	D	[(0.667, 1, 1.5), (0.55, 0.7, 0.85)]	0.3	(0.14, 0.17, 0.21)
Freight Cost	S	[(2.5, 3, 3.5), (0.65, 0.8, 0.95)]	–	(0.42, 0.48, 0.54)
	O	[(1.5, 2, 2.5), (0.55, 0.7, 0.85)]	0.45	(0.32, 0.35, 0.39)
	D	[(0.667, 1, 1.5), (0.45, 0.6, 0.75)]	0.35	(0.15, 0.18, 0.20)

**Table 5:** Z-WASPAS weighted sum model (WSM) and weighted product model (WPM) values for identified failure modes.

Failure mode	Objective	WSM TFN	WPM TFN	Combined (K)
F01	Delivery to Client	(0.264, 0.311, 0.472)	(0.153, 0.276, 0.541)	(0.209, 0.293, 0.507)
	Freight Cost	(0.309, 0.377, 0.535)	(0.236, 0.374, 0.641)	(0.273, 0.376, 0.588)
F02	Delivery to Client	(0.362, 0.437, 0.622)	(0.223, 0.388, 0.678)	(0.292, 0.413, 0.650)
	Freight Cost	(0.319, 0.410, 0.684)	(0.242, 0.400, 0.763)	(0.281, 0.405, 0.723)
F03	Delivery to Client	(0.278, 0.323, 0.431)	(0.155, 0.274, 0.476)	(0.216, 0.299, 0.453)
	Freight Cost	(0.292, 0.360, 0.487)	(0.218, 0.352, 0.585)	(0.255, 0.356, 0.536)
F04	Delivery to Client	(0.201, 0.220, 0.294)	(0.097, 0.164, 0.277)	(0.149, 0.192, 0.285)
	Freight Cost	(0.193, 0.220, 0.276)	(0.129, 0.198, 0.315)	(0.161, 0.209, 0.296)
F05	Delivery to Client	(0.263, 0.303, 0.396)	(0.152, 0.268, 0.465)	(0.208, 0.285, 0.431)
	Freight Cost	(0.328, 0.421, 0.608)	(0.249, 0.413, 0.708)	(0.288, 0.417, 0.658)
F06	Delivery to Client	(0.293, 0.348, 0.494)	(0.156, 0.278, 0.493)	(0.224, 0.313, 0.493)
	Freight Cost	(0.296, 0.360, 0.485)	(0.224, 0.359, 0.599)	(0.260, 0.359, 0.542)
F07	Delivery to Client	(0.352, 0.437, 0.693)	(0.214, 0.384, 0.728)	(0.283, 0.411, 0.711)
	Freight Cost	(0.327, 0.420, 0.633)	(0.249, 0.412, 0.736)	(0.288, 0.416, 0.684)
F08	Delivery to Client	(0.302, 0.358, 0.508)	(0.161, 0.285, 0.504)	(0.232, 0.322, 0.506)
	Freight Cost	(0.252, 0.304, 0.393)	(0.187, 0.301, 0.495)	(0.220, 0.302, 0.444)
F09	Delivery to Client	(0.320, 0.384, 0.555)	(0.194, 0.347, 0.634)	(0.257, 0.366, 0.595)
	Freight Cost	(0.266, 0.329, 0.443)	(0.193, 0.313, 0.519)	(0.229, 0.321, 0.481)
F10	Delivery to Client	(0.271, 0.315, 0.419)	(0.157, 0.277, 0.484)	(0.214, 0.296, 0.452)
	Freight Cost	(0.351, 0.451, 0.665)	(0.269, 0.441, 0.766)	(0.310, 0.446, 0.716)
F11	Delivery to Client	(0.279, 0.328, 0.458)	(0.163, 0.290, 0.514)	(0.221, 0.309, 0.486)
	Freight Cost	(0.307, 0.380, 0.540)	(0.228, 0.367, 0.630)	(0.268, 0.374, 0.585)
F12	Delivery to Client	(0.359, 0.446, 0.696)	(0.218, 0.393, 0.731)	(0.288, 0.419, 0.714)
	Freight Cost	(0.330, 0.424, 0.637)	(0.252, 0.417, 0.743)	(0.291, 0.421, 0.690)
F13	Delivery to Client	(0.176, 0.186, 0.212)	(0.090, 0.155, 0.251)	(0.133, 0.171, 0.232)
	Freight Cost	(0.206, 0.232, 0.278)	(0.144, 0.220, 0.343)	(0.175, 0.226, 0.310)
F14	Delivery to Client	(0.157, 0.159, 0.175)	(0.076, 0.127, 0.203)	(0.116, 0.143, 0.189)
	Freight Cost	(0.182, 0.200, 0.228)	(0.124, 0.188, 0.286)	(0.153, 0.194, 0.257)
F15	Delivery to Client	(0.305, 0.359, 0.495)	(0.181, 0.319, 0.565)	(0.243, 0.339, 0.530)
	Freight Cost	(0.259, 0.308, 0.390)	(0.191, 0.302, 0.486)	(0.225, 0.305, 0.438)
F16	Delivery to Client	(0.199, 0.206, 0.248)	(0.104, 0.171, 0.285)	(0.152, 0.188, 0.266)
	Freight Cost	(0.169, 0.185, 0.217)	(0.114, 0.171, 0.266)	(0.141, 0.178, 0.242)
F17	Delivery to Client	(0.255, 0.293, 0.369)	(0.145, 0.257, 0.433)	(0.200, 0.275, 0.401)
	Freight Cost	(0.321, 0.387, 0.519)	(0.245, 0.384, 0.632)	(0.283, 0.386, 0.576)
F18	Delivery to Client	(0.280, 0.333, 0.469)	(0.162, 0.291, 0.526)	(0.221, 0.312, 0.497)
	Freight Cost	(0.290, 0.353, 0.471)	(0.214, 0.341, 0.557)	(0.252, 0.347, 0.514)
F19	Delivery to Client	(0.354, 0.439, 0.643)	(0.217, 0.390, 0.700)	(0.285, 0.414, 0.672)
	Freight Cost	(0.343, 0.434, 0.629)	(0.260, 0.420, 0.722)	(0.301, 0.427, 0.675)
F20	Delivery to Client	(0.207, 0.221, 0.254)	(0.110, 0.187, 0.300)	(0.159, 0.204, 0.277)
	Freight Cost	(0.185, 0.208, 0.258)	(0.123, 0.185, 0.291)	(0.154, 0.197, 0.274)
F21	Delivery to Client	(0.263, 0.305, 0.394)	(0.151, 0.266, 0.454)	(0.207, 0.286, 0.424)
	Freight Cost	(0.328, 0.403, 0.561)	(0.251, 0.399, 0.671)	(0.290, 0.401, 0.616)

**Table 6:** Hybrid rankings for delivery to client date using Z-SWARA, Z-WASPAS, and Z-DEA-FMEA.

Factor	Z-WASPAS combined K TFN	Z-DEA efficiency (θ)	RPN middle value	Hybrid score (Q_i_)	Rank
F01	(0.209, 0.293, 0.507)	0.6341	109.44	0.464874	12
F02	(0.292, 0.413, 0.650)	0.6929	94.5	0.325713	18
F03	(0.216, 0.299, 0.453)	0.6542	93.6	0.449661	14
F04	(0.149, 0.192, 0.285)	0.5536	163.2	0.609799	5
F05	(0.208, 0.285, 0.431)	0.6554	110	0.482154	9
F06	(0.224, 0.313, 0.493)	0.7303	101.25	0.465203	11
F07	(0.283, 0.411, 0.711)	0.6739	72	0.300962	19
F08	(0.232, 0.322, 0.506)	0.6667	70	0.404198	15
F09	(0.257, 0.366, 0.595)	0.6255	63	0.330843	17
F10	(0.214, 0.296, 0.452)	0.6647	108	0.470117	10
F11	(0.221, 0.309, 0.486)	0.7345	113.75	0.483008	8
F12	(0.288, 0.419, 0.714)	0.6857	69.3	0.292733	20
F13	(0.133, 0.171, 0.232)	0.6813	236.25	0.745367	2
F14	(0.116, 0.143, 0.189)	1	360	1	1
F15	(0.243, 0.339, 0.530)	0.5783	63	0.347718	16
F16	(0.152, 0.188, 0.266)	0.6666	243.36	0.726519	3
F17	(0.200, 0.275, 0.401)	0.6832	126	0.518313	6
F18	(0.221, 0.312, 0.497)	0.681	101.25	0.449977	13
F19	(0.285, 0.414, 0.672)	0.6643	70.4	0.292657	21
F20	(0.159, 0.204, 0.277)	0.6636	210	0.675306	4
F21	(0.207, 0.286, 0.424)	0.6731	113.4	0.489995	7

**Table 7:** Hybrid rankings for freight cost using Z-SWARA, Z-WASPAS, and Z-DEA-FMEA.

Factor	Z-WASPAS combined K TFN	Z-DEA efficiency (θ)	RPN middle value	Hybrid score (Q_i_)	Rank
F01	(0.273, 0.376, 0.588)	0.5998	62.4	0.359503	15
F02	(0.281, 0.405, 0.723)	0.6645	78.4	0.363592	14
F03	(0.255, 0.356, 0.536)	0.5847	66.15	0.383703	13
F04	(0.161, 0.209, 0.296)	0.6881	184.45	0.738464	3
F05	(0.288, 0.417, 0.658)	0.6749	64.512	0.335996	17
F06	(0.260, 0.359, 0.542)	0.6242	75.6	0.404119	10
F07	(0.288, 0.416, 0.684)	0.6525	56	0.319882	19
F08	(0.220, 0.302, 0.444)	0.7757	103.95	0.558455	6
F09	(0.229, 0.321, 0.481)	0.5516	67.2	0.417422	8
F10	(0.310, 0.446, 0.716)	0.7296	57.6	0.310128	21
F11	(0.268, 0.374, 0.585)	0.7047	63	0.397655	11
F12	(0.291, 0.421, 0.690)	0.6467	56	0.31173	20
F13	(0.175, 0.226, 0.310)	0.6468	187.2	0.706748	4
F14	(0.153, 0.194, 0.257)	0.6838	286.875	0.8747	2
F15	(0.225, 0.305, 0.438)	0.7417	92.4	0.52997	7
F16	(0.141, 0.178, 0.242)	1	252	0.959477	1
F17	(0.283, 0.386, 0.576)	0.6496	81	0.385278	12
F18	(0.252, 0.347, 0.514)	0.5807	78.75	0.408204	9
F19	(0.301, 0.427, 0.675)	0.7206	64	0.338196	16
F20	(0.154, 0.197, 0.274)	0.6471	149.5	0.699112	5
F21	(0.290, 0.401, 0.616)	0.6013	63	0.329606	18

**Table 8:** Combined hybrid rankings for HIV drug supply chain failure modes using Z-SWARA, Z-WASPAS, and Z-DEA-FMEA.

Factor	Total hybrid score (Q_total_)	Total rank
F14	0.93735	1
F16	0.842998	2
F13	0.726058	3
F20	0.687209	4
F04	0.674131	5
F08	0.481326	6
F17	0.451796	7
F11	0.440331	8
F15	0.438844	9
F06	0.434661	10
F18	0.429091	11
F03	0.416682	12
F01	0.412189	13
F21	0.409801	14
F05	0.409075	15
F10	0.390123	16
F09	0.374132	17
F02	0.344653	18
F19	0.315426	19
F07	0.310422	20
F12	0.302231	21

Step 1: According to their judgment, professionals rank factors from most important to least important in descending order.

Step 2: Experts should assess each criterion’s linguistic quality according to its relevance to j−1 , based on their first judgment. According to Table 9, the quantity of the first component ( ${\tilde{F}}_{j}$ ) can be calculated. To calculate the dependability component ( ${\tilde{L}}_{j}$ ), it is necessary to refer to Table 10. Each circumstance is assigned a Z-number.

**Table 9:** Linguistic quality plays a significant role in weighting.

Linguistic variables	TFNs
Equality of importance (EI)	(1, 1, 1)
Modestly less important (MOL)	(2/3, 1, 3/2)
Least important (LI)	(2/5, 1/2, 2/3)
Very low importance (VLI)	(2/7, 1/3, 2/5)
Much less important (MUL)	(2/9, 1/4, 2/7)

**Table 10:** Assessing reliability using linguistic variables.

Linguistic variables	Very weak (VW)	Weak (W)	Medium (M)	High (H)	Very high (VH)
TFNs	(0,0,0.25)	(0.2,0.35,0.5)	(0.35,0.5,0.75)	(0.5,0.75,0.9)	(0.75,1,1)

Step 3: The Z-number from Step 2 is converted to a TFN using Equations 5, 6 (67). For example, a Z-number [(2/7, 1/3, 2/5), (0.35, 0.5, 0.75)] (VLI, M) is transformed into (0.21, 0.24, 0.29), as shown in Table 11.

**Table 11:** In order to determine weighting criteria, Z-numbers are transformed to TFNs based on linguistic characteristics.

Linguistics variables	TFNs	Linguistics variables	TFNs
(EI, VW)	(1, 1, 1)	(LI, H)	(0.34, 0.42, 0.56)
(EI, W)	(1, 1, 1)	(LI, VH)	(0.38, 0.48, 0.64)
(EI, M)	(1, 1, 1)	(VLI, VW)	(0.08, 0.10, 0.12)
(EI, H)	(1, 1, 1)	(VLI, W)	(0.17, 0.20, 0.24)
(EI, VH)	(1, 1, 1)	(VLI, M)	(0.21, 0.24, 0.29)
(MOL, VW)	(0.19, 0.29, 0.43)	(VLI, H)	(0.24, 0.28, 0.34)
(MOL, W)	(0.39, 0.59, 0.89)	(VLI, VH)	(0.27, 0.32, 0.38)
(MOL, M)	(0.49, 0.73, 1.10)	(MUL, VW)	(0.06, 0.07, 0.08)
(MOL, H)	(0.56, 0.85, 1.27)	(MUL, W)	(0.13, 0.15, 0.17)
(MOL, VH)	(0.64, 0.96, 1.44)	(MUL, M)	(0.16, 0.18, 0.21)
(LI, VW)	(0.12, 0.14, 0.19)	(MUL, H)	(0.19, 0.21, 0.24)
(LI, W)	(0.24, 0.30, 0.39)	(MUL, VH)	(0.21, 0.24, 0.27)
(LI, M)	(0.29, 0.37, 0.49)		

Step 4: Compute coefficients $q_{j}$ for each criterion, starting with 𝑞_𝑆_ = (1,1,1). For subsequent criteria, ${\tilde{q}}_{j}$ is calculated as Equation 7:


$$q_{j}=\frac{F_{j}}{F_{j-1}+1}$$ (7)

where $F_{j}$ is TFN from step 3, reflecting relative importance to the prior criterion.

Step 5: Normalize these coefficients into final weights using Equation 8:


$$w_{j}=\frac{q_{j}}{\overset{n}{\underset{j=1}{\sum }}q_{j}}$$ (8)

where $w_{j}$ is a TFN.

### 3.5 Z-WASPAS method

The Weighted Aggregated Sum Product Assessment (WASPAS) method, introduced by Zavadskas et al. (70), combines Weighted Sum Model (WSM) and Weighted Product Model (WPM) scores for multi-criteria decision-making. Fuzzy WASPAS extends this by using TFNs to handle uncertainty (71). In this study, we further extend fuzzy WASPAS to Z-WASPAS by using Z-SWARA weights (Table 4), which incorporate reliability via Z-numbers (69). Z-WASPAS ranks failure modes based on their risk impact (Severity, Occurrence, Detection), with WSM and WPM scores (Table 5) used in Z-DEA-FMEA (Table 12) and hybrid rankings (Tables 6–8). The Z-WASPAS method involves the following steps:

**Table 12:** Z-DEA-FMEA input and output averages for HIV drug supply chain failure modes.

Failure mode	Objective	S_avg_	O_avg_	$\frac{1}{K_{avg}}$
F01	Delivery to Client	2.637	3.68	2.973
F01	Freight Cost	3.04	2.802	2.425
F02	Delivery to Client	1.86	2.643	2.214
F02	Freight Cost	2.217	2.625	2.129
F03	Delivery to Client	3.6	2.6	3.099
F03	Freight Cost	2.987	3.825	2.616
F04	Delivery to Client	7.067	5.5	4.792
F04	Freight Cost	7.792	4.752	4.505
F05	Delivery to Client	3	3.5	3.247
F05	Freight Cost	2.123	3.2	2.201
F06	Delivery to Client	3.6	2.1	2.913
F06	Freight Cost	2.8	3.375	2.584
F07	Delivery to Client	1.65	3.2	2.135
F07	Freight Cost	2.625	2.123	2.161
F08	Delivery to Client	4.25	2.1	2.83
F08	Freight Cost	3.2	3.6	3.106
F09	Delivery to Client	2.275	3	2.463
F09	Freight Cost	4.25	3.413	2.91
F10	Delivery to Client	2.8	3.375	3.119
F10	Freight Cost	1.96	2.8	2.038
F11	Delivery to Client	2.275	4.25	2.953
F11	Freight Cost	3.6	2.1	2.445
F12	Delivery to Client	1.65	2.987	2.111
F12	Freight Cost	2.625	2.123	2.14
F13	Delivery to Client	6.25	6.183	5.597
F13	Freight Cost	5.692	5.306	4.219
F14	Delivery to Client	8.097	7.067	6.696
F14	Freight Cost	7.083	5.875	4.967
F15	Delivery to Client	2.8	3.375	2.698
F15	Freight Cost	4	2.773	3.099
F16	Delivery to Client	5.953	4.825	4.95
F16	Freight Cost	8.097	6.183	5.348
F17	Delivery to Client	3.2	3.5	3.425
F17	Freight Cost	2.925	2.4	2.41
F18	Delivery to Client	2.45	3.375	2.913
F18	Freight Cost	4	2.773	2.695
F19	Delivery to Client	1.797	2.8	2.188
F19	Freight Cost	2.6	1.96	2.138
F20	Delivery to Client	5.25	4.8	4.688
F20	Freight Cost	8.097	5.417	4.8
F21	Delivery to Client	3.15	3.2	3.272
F21	Freight Cost	2.6	3.15	2.295

Step 1: Use the $\tilde{\bar{H}}\;$ matrices to normalize the non-beneficial components (67, 70) (Equation 9).


$${\hat{h}}_{ij}=\frac{min_{i}{\bar{h}}_{ij}}{{\bar{h}}_{ij}}$$ (9)

This ensures the least risky mode (lowest S, O, D) approaches (1,1,1), with riskier modes scoring <(1,1,1), aligning with FMEA risk prioritization.

Step 2: Calculate the following matrix to evaluate normalized fuzzy choices of ${\hat{h}}_{ij}\;$ made by the WSM ( $\tilde{Q}\;$ TFN) and WPM ( $\tilde{P}\;$ TFN) using Equations 10, 11:


$${\tilde{Q}}_{i}=\overset{n}{\underset{j=1}{\sum }}{\hat{h}}_{ij}{\tilde{w}}_{j}$$ (10)

$${\tilde{P}}_{i}=\overset{n}{\underset{j=1}{\prod }}{\hat{h}}_{ij}{\tilde{w}}_{j}$$ (11)

Examine each TFN’s region of decision-making to de-fuzzify performance assessment Equations 12–14:


$${\bar{Q}}_{i}=\frac{1}{3}\left(a_{i}^{Q}+b_{i}^{Q}+c_{i}^{Q}\right)$$ (12)

$${\bar{P}}_{i}=\frac{1}{3}\left(a_{i}^{P}+b_{i}^{P}+c_{i}^{p}\right)$$ (13)

$$K_{i}=\lambda \cdot {\bar{Q}}_{i}+\left(1-\lambda \right)\;\cdot \;{\bar{P}}_{i}$$

0≤λ≤1 (14)

Step 3: To order the failure modes for the 𝑖-th alternative, use the utility function Q_i_, where 𝜆 (typically set to 0.5 for balance) weights the WSM and WPM scores. Rank the failure modes from highest to lowest K_𝑖_ values to determine their priority (lower middle values indicate higher risk).

### 3.6 Data Envelopment Analysis (DEA) in Z-DEA-FMEA

In this study, the Z-DEA-FMEA model evaluates the efficiency of failure modes in the HIV drug supply chain, leveraging fuzzy Z-number attributes processed through a linear programming (LP) framework (58, 71) Z-DEA-FMEA assesses the efficiency of 42 failure modes (21 modes × 2 objectives, Delivery to Client Date and Freight Cost).

Inputs (Severity 𝑆, Occurrence 𝑂) and output (reciprocal Z-WASPAS 1/𝐾_avg_) are derived as Triangular Fuzzy Numbers (TFNs) from Z-numbers in Table 13. Table 13 presents Z-numbers for S, O, and D, derived from structured interviews with drug supply chain and HIV drug logistics experts. Experts rated these factors on a 1–10 scale as Z-numbers, reflecting severity, occurrence, and detection with associated reliability. Individual responses were aggregated by averaging, producing the Z-numbers shown, which were then converted to TFNs using Equations 5, 6 for analysis in Z-SWARA, Z-WASPAS, and Z-DEA-FMEA. Minor rounding was applied during aggregation for consistency, ensuring analytical precision. To enable crisp LP, TFNs are de-fuzzified into averages using Equation 15:


$$\begin{array}{c} x_{ij}^{avg}=\frac{l_{ij}+m_{ij}+u_{ij}}{3}\; \end{array}$$ (15)

where 𝑥_𝑖𝑗_ is the 𝑖-th input (S or O) for mode 𝑗, and 𝑦_𝑗_ is the output (𝐾), with 𝑙, 𝑚, and 𝑢 as lower, middle, and upper TFN bounds. These averages (𝑆_𝑎𝑣𝑔_, 𝑂_𝑎𝑣𝑔_, 𝐾_𝑎𝑣𝑔_) are detailed in Table 12 for all modes. This study uses 1/𝐾_avg_ scores as the output (low 𝐾_𝑎𝑣𝑔_ values indicate high risk due to non-beneficial criteria S, O, D), so high 1/𝐾_𝑎𝑣𝑔_ values reflect greater criticality) for Z-DEA-FMEA. To achieve robust differentiation among failure modes, super-efficiency DEA with Variable Returns to Scale (VRS) was employed using the “sdea()” function in “RStudio” (72). This method enhances standard DEA by allowing efficiency scores to exceed 1 for highly efficient modes, addressing the need for clear prioritization in supply chain risk management (73). The super-efficiency model is formulated as Equations 16–20:


$$\theta_{j}=\begin{array}{c} \max\theta_{j} \end{array}$$ (16)

**Table 13:** Z-numbers describing the risk factors associated with identified failure modes for Delivery to Client Date and Freight Cost (USD).

Failure mode	Objective	S Z-number	O Z-number	D Z-number
F01	Delivery to Client	[(2.0, 3.8, 5.5), (0.5, 0.7, 0.9)]	[(3.0, 4.8, 6.0), (0.6, 0.8, 1.0)]	[(4.0, 6.0, 8.0), (0.55, 0.75, 0.95)]
F01	Freight Cost	[(3.5, 5.2, 6.5), (0.4, 0.6, 0.8)]	[(2.5, 4.0, 5.8), (0.5, 0.7, 0.85)]	[(1.5, 3.0, 4.5), (0.6, 0.8, 1.0)]
F02	Delivery to Client	[(1.8, 3.0, 4.5), (0.45, 0.6, 0.75)]	[(2.5, 4.2, 5.5), (0.5, 0.65, 0.8)]	[(6.0, 7.5, 9.0), (0.4, 0.55, 0.7)]
F02	Freight Cost	[(1.5, 3.2, 4.8), (0.5, 0.7, 0.9)]	[(2.0, 3.5, 5.0), (0.55, 0.75, 0.95)]	[(5.5, 7.0, 8.5), (0.45, 0.6, 0.8)]
F03	Delivery to Client	[(3.0, 4.5, 6.0), (0.65, 0.8, 0.95)]	[(2.5, 4.0, 5.5), (0.5, 0.65, 0.8)]	[(3.8, 5.2, 6.8), (0.6, 0.75, 0.9)]
F03	Freight Cost	[(2.8, 4.2, 5.8), (0.55, 0.7, 0.85)]	[(3.0, 4.5, 6.0), (0.7, 0.85, 1.0)]	[(2.0, 3.5, 5.0), (0.45, 0.6, 0.75)]
F04	Delivery to Client	[(6.5, 8.0, 9.5), (0.75, 0.9, 1.0)]	[(5.0, 6.8, 8.0), (0.7, 0.85, 0.95)]	[(1.5, 3.0, 4.5), (0.5, 0.65, 0.8)]
F04	Freight Cost	[(7.0, 8.5, 10.0), (0.8, 0.95, 1.0)]	[(4.5, 6.2, 7.5), (0.65, 0.8, 0.9)]	[(2.0, 3.5, 5.0), (0.55, 0.7, 0.85)]
F05	Delivery to Client	[(2.5, 4.0, 5.5), (0.6, 0.75, 0.9)]	[(3.5, 5.0, 6.5), (0.55, 0.7, 0.85)]	[(4.0, 5.5, 7.0), (0.65, 0.8, 0.95)]
F05	Freight Cost	[(1.8, 3.2, 4.8), (0.5, 0.65, 0.8)]	[(2.8, 4.2, 5.8), (0.6, 0.75, 0.9)]	[(3.0, 4.5, 6.0), (0.55, 0.7, 0.85)]
F06	Delivery to Client	[(3.0, 4.5, 6.0), (0.65, 0.8, 0.95)]	[(2.0, 3.5, 5.0), (0.45, 0.6, 0.75)]	[(5.0, 6.5, 8.0), (0.7, 0.85, 1.0)]
F06	Freight Cost	[(2.5, 4.0, 5.5), (0.55, 0.7, 0.85)]	[(3.0, 4.5, 6.0), (0.6, 0.75, 0.9)]	[(2.8, 4.2, 5.8), (0.5, 0.65, 0.8)]
F07	Delivery to Client	[(1.5, 3.0, 4.5), (0.4, 0.55, 0.7)]	[(2.5, 4.0, 5.5), (0.65, 0.8, 0.95)]	[(4.5, 6.0, 7.5), (0.55, 0.7, 0.85)]
F07	Freight Cost	[(2.0, 3.5, 5.0), (0.6, 0.75, 0.9)]	[(1.8, 3.2, 4.8), (0.5, 0.65, 0.8)]	[(3.5, 5.0, 6.5), (0.6, 0.75, 0.9)]
F08	Delivery to Client	[(3.5, 5.0, 6.5), (0.7, 0.85, 1.0)]	[(2.0, 3.5, 5.0), (0.45, 0.6, 0.75)]	[(2.5, 4.0, 5.5), (0.55, 0.7, 0.85)]
F08	Freight Cost	[(2.8, 4.2, 5.8), (0.6, 0.75, 0.9)]	[(3.0, 4.5, 6.0), (0.65, 0.8, 0.95)]	[(4.0, 5.5, 7.0), (0.7, 0.85, 1.0)]
F09	Delivery to Client	[(2.0, 3.5, 5.0), (0.5, 0.65, 0.8)]	[(2.5, 4.0, 5.5), (0.6, 0.75, 0.9)]	[(3.0, 4.5, 6.0), (0.55, 0.7, 0.85)]
F09	Freight Cost	[(3.5, 5.0, 6.5), (0.7, 0.85, 1.0)]	[(2.8, 4.2, 5.8), (0.65, 0.8, 0.95)]	[(1.8, 3.2, 4.8), (0.45, 0.6, 0.75)]
F10	Delivery to Client	[(2.5, 4.0, 5.5), (0.55, 0.7, 0.85)]	[(3.0, 4.5, 6.0), (0.6, 0.75, 0.9)]	[(4.5, 6.0, 7.5), (0.65, 0.8, 0.95)]
F10	Freight Cost	[(1.8, 3.2, 4.8), (0.45, 0.6, 0.75)]	[(2.5, 4.0, 5.5), (0.55, 0.7, 0.85)]	[(3.0, 4.5, 6.0), (0.6, 0.75, 0.9)]
F11	Delivery to Client	[(2.0, 3.5, 5.0), (0.5, 0.65, 0.8)]	[(3.5, 5.0, 6.5), (0.7, 0.85, 1.0)]	[(5.0, 6.5, 8.0), (0.55, 0.7, 0.85)]
F11	Freight Cost	[(3.0, 4.5, 6.0), (0.65, 0.8, 0.95)]	[(2.0, 3.5, 5.0), (0.45, 0.6, 0.75)]	[(2.5, 4.0, 5.5), (0.6, 0.75, 0.9)]
F12	Delivery to client	[(1.5, 3.0, 4.5), (0.4, 0.55, 0.7)]	[(2.8, 4.2, 5.8), (0.55, 0.7, 0.85)]	[(4.0, 5.5, 7.0), (0.65, 0.8, 0.95)]
F12	Freight cost	[(2.0, 3.5, 5.0), (0.6, 0.75, 0.9)]	[(1.8, 3.2, 4.8), (0.5, 0.65, 0.8)]	[(3.5, 5.0, 6.5), (0.55, 0.7, 0.85)]
F13	Delivery to Client	[(6.0, 7.5, 9.0), (0.7, 0.85, 0.95)]	[(5.5, 7.0, 8.5), (0.75, 0.9, 1.0)]	[(3.0, 4.5, 6.0), (0.6, 0.75, 0.9)]
F13	Freight Cost	[(5.8, 7.2, 8.8), (0.65, 0.8, 0.9)]	[(4.8, 6.5, 7.8), (0.7, 0.85, 0.95)]	[(2.5, 4.0, 5.5), (0.5, 0.65, 0.8)]
F14	Delivery to Client	[(7.5, 9.0, 10.0), (0.8, 0.95, 1.0)]	[(6.5, 8.0, 9.5), (0.75, 0.9, 1.0)]	[(3.5, 5.0, 6.5), (0.55, 0.7, 0.85)]
F14	Freight Cost	[(7.0, 8.5, 10.0), (0.7, 0.85, 0.95)]	[(6.0, 7.5, 9.0), (0.65, 0.8, 0.9)]	[(3.0, 4.5, 6.0), (0.5, 0.65, 0.8)]
F15	Delivery to Client	[(2.5, 4.0, 5.5), (0.55, 0.7, 0.85)]	[(3.0, 4.5, 6.0), (0.6, 0.75, 0.9)]	[(2.0, 3.5, 5.0), (0.45, 0.6, 0.75)]
F15	Freight Cost	[(3.5, 5.0, 6.5), (0.65, 0.8, 0.95)]	[(2.8, 4.2, 5.8), (0.5, 0.65, 0.8)]	[(4.0, 5.5, 7.0), (0.6, 0.75, 0.9)]
F16	Delivery to Client	[(6.0, 7.8, 9.0), (0.65, 0.8, 0.9)]	[(4.8, 6.5, 8.0), (0.6, 0.75, 0.9)]	[(3.0, 4.8, 6.0), (0.55, 0.7, 0.85)]
F16	Freight Cost	[(7.5, 9.0, 10.0), (0.8, 0.95, 1.0)]	[(5.5, 7.0, 8.5), (0.75, 0.9, 1.0)]	[(2.5, 4.0, 5.5), (0.6, 0.75, 0.9)]
F17	Delivery to Client	[(2.8, 4.2, 5.8), (0.6, 0.75, 0.9)]	[(3.5, 5.0, 6.5), (0.55, 0.7, 0.85)]	[(4.5, 6.0, 7.5), (0.65, 0.8, 0.95)]
F17	Freight Cost	[(3.0, 4.5, 6.0), (0.5, 0.65, 0.8)]	[(2.5, 4.0, 5.5), (0.45, 0.6, 0.75)]	[(3.0, 4.5, 6.0), (0.55, 0.7, 0.85)]
F18	Delivery to Client	[(2.0, 3.5, 5.0), (0.55, 0.7, 0.85)]	[(3.0, 4.5, 6.0), (0.6, 0.75, 0.9)]	[(5.0, 6.5, 8.0), (0.7, 0.85, 1.0)]
F18	Freight Cost	[(3.5, 5.0, 6.5), (0.65, 0.8, 0.95)]	[(2.8, 4.2, 5.8), (0.5, 0.65, 0.8)]	[(2.0, 3.5, 5.0), (0.45, 0.6, 0.75)]
F19	Delivery to Client	[(1.8, 3.2, 4.8), (0.4, 0.55, 0.7)]	[(2.5, 4.0, 5.5), (0.55, 0.7, 0.85)]	[(4.0, 5.5, 7.0), (0.6, 0.75, 0.9)]
F19	Freight Cost	[(2.5, 4.0, 5.5), (0.5, 0.65, 0.8)]	[(1.8, 3.2, 4.8), (0.45, 0.6, 0.75)]	[(3.5, 5.0, 6.5), (0.65, 0.8, 0.95)]
F20	Delivery to Client	[(5.5, 7.0, 8.5), (0.6, 0.75, 0.9)]	[(4.5, 6.0, 7.5), (0.65, 0.8, 0.95)]	[(3.5, 5.0, 6.5), (0.5, 0.65, 0.8)]
F20	Freight Cost	[(7.5, 9.0, 10.0), (0.8, 0.95, 1.0)]	[(5.0, 6.5, 8.0), (0.7, 0.85, 0.95)]	[(2.0, 3.5, 5.0), (0.55, 0.7, 0.85)]
F21	Delivery to Client	[(3.0, 4.5, 6.0), (0.55, 0.7, 0.85)]	[(2.8, 4.2, 5.8), (0.6, 0.75, 0.9)]	[(4.5, 6.0, 7.5), (0.65, 0.8, 0.95)]
F21	Freight Cost	[(2.5, 4.0, 5.5), (0.5, 0.65, 0.8)]	[(3.0, 4.5, 6.0), (0.55, 0.7, 0.85)]	[(2.0, 3.5, 5.0), (0.45, 0.6, 0.75)]

Subject to:


$$\begin{array}{c} \overset{n}{\underset{k=1}{\sum }}\lambda_{k}\cdot y_{k}^{avg}\geq \theta_{j}\cdot y_{j}^{avg} \end{array}$$ (17)

$$\begin{array}{c} \overset{n}{\underset{k=1}{\sum }}\lambda_{k}\cdot x_{ik}^{avg}\leq x_{ij}^{avg},i=S,O \end{array}$$ (18)

$$\begin{array}{c} \overset{n}{\underset{k=1}{\sum }}\lambda_{k}=1 \end{array}$$ (19)

$$\lambda_{k}\geq 0$$ (20)

Here, 𝜆_𝑘_ are weights for peer modes 𝑘 (42 total), ensuring Variable Returns to Scale (VRS) (73). Results are compiled in Table 14 for use in subsequent hybrid rankings, adapting traditional DEA to incorporate Z-number uncertainty (58).

**Table 14:** Normalized super-efficiency scores for HIV drug supply chain failure modes across delivery and freight objectives.

Failure mode	Delivery efficiency ( $\theta_{j}^{norm}$ )	Freight efficiency ( $\theta_{j}^{norm}$ )
F01	0.6341	0.5998
F02	0.6929	0.6645
F03	0.6542	0.5847
F04	0.5536	0.6881
F05	0.6554	0.6749
F06	0.7303	0.6242
F07	0.6739	0.6525
F08	0.6667	0.7757
F09	0.6255	0.5516
F10	0.6647	0.7296
F11	0.7345	0.7047
F12	0.6857	0.6467
F13	0.6813	0.6468
F14	1	0.6838
F15	0.5783	0.7417
F16	0.6666	1
F17	0.6832	0.6496
F18	0.681	0.5807
F19	0.6643	0.7206
F20	0.6636	0.6471
F21	0.6731	0.6013

As a common practice in DEA (72), super-efficiency scores producing infinite values for outliers (e.g., F14) were capped at 1.5 to ensure usability and prevent instability, then normalized relative to 1.5. This preserves rankings, enhancing compatibility with Z-SWARA and Z-WASPAS outputs (74).

Our Z-DEA-FMEA extension is informed by DEA applications in hospital efficiency. In their evaluation of Portuguese hospitals before and after COVID-19, Nunes and Ferreira (32) employed network DEA and discovered that, while efficiency decreased in 2020, recoveries surpassed pre-pandemic levels with enhanced safety, proving the importance of DEA for disruption resilience. Applying log-linear DEA for pay-for-performance optimization, Ferreira et al. (33) were able to link payments with efficiency and quality, which is pertinent to our freight cost risks, and achieve 30% cost savings. The DEA’s robustness in healthcare under uncertainty is highlighted by these and other research (27, 29, 31).

### 3.7 Hybrid rankings

This section outlines the hybrid ranking methodology, which integrates Z-DEA-FMEA with Z-SWARA and Z-WASPAS to provide a comprehensive prioritization of failure modes across two objectives: Delivery to Client Date and Freight Cost (74). The process combines three rankings: RPN (Table 15), Z-SWARA/Z-WASPAS (Table 5), and Z-DEA-FMEA efficiencies (Table 14). The scores are normalized and aggregated into a hybrid score $Q_{i}$ for each failure mode 𝑖 using Equation 21:


$$\begin{array}{c} \begin{array}{c} Q_{ij}=\frac{1}{3}\cdot \mathrm{Norm}.{\mathrm{RPN}}_{i}+\frac{1}{3}\cdot \mathrm{Norm}.K_{ij}+\frac{1}{3}\cdot \mathrm{Norm}.\theta_{j} \end{array} \end{array}$$ (21)

where $\mathrm{Norm}.{\mathrm{RPN}}_{i}$ is the normalized RPN middle value (Table 15, divided by the maximum RPN), $\mathrm{Norm}.K_{ij}$ is the Z-WASPAS middle score, normalized across its range as $Norm.K_{ij}=1-\frac{K_{\mathrm{middle}}-\min K_{\mathrm{middle}}}{\max\left(K_{\mathrm{middle}}\right)-\min K_{\mathrm{middle}}}$ (Table 5, reflecting higher risk with lower 𝐾_𝑎𝑣𝑔_) and $\theta_{j}$ is the Z-DEA-FMEA efficiency (Table 14).

**Table 15:** Risk priority numbers (RPNs) for identified failure modes.

Failure mode	Objective	RPN (TFN)
F01	Delivery to Client	(24.00, 109.44, 264.00)
F01	Freight Cost	(13.125, 62.40, 169.65)
F02	Delivery to Client	(27.00, 94.50, 222.75)
F02	Freight Cost	(16.50, 78.40, 204.00)
F03	Delivery to Client	(28.50, 93.60, 224.40)
F03	Freight Cost	(16.80, 66.15, 174.00)
F04	Delivery to Client	(48.75, 163.20, 342.00)
F04	Freight Cost	(63.00, 184.45, 375.00)
F05	Delivery to Client	(35.00, 110.00, 250.25)
F05	Freight Cost	(15.12, 64.512, 167.04)
F06	Delivery to Client	(30.00, 102.375, 240.00)
F06	Freight Cost	(21.00, 75.60, 191.40)
F07	Delivery to Client	(16.875, 72.0, 185.625)
F07	Freight Cost	(12.6, 56.0, 156.0)
F08	Delivery to Client	(17.5, 70.0, 178.75)
F08	Freight Cost	(33.6, 103.95, 243.6)
F09	Delivery to Client	(15.0, 63.0, 165.0)
F09	Freight Cost	(17.64, 67.20, 180.96)
F10	Delivery to Client	(33.75, 108.0, 247.5)
F10	Freight Cost	(13.5, 57.60, 158.4)
F11	Delivery to Client	(35.0, 113.75, 260.0)
F11	Freight Cost	(15.0, 63.0, 165.0)
F12	Delivery to Client	(16.8, 69.30, 182.7)
F12	Freight Cost	(12.6, 56.0, 156.0)
F13	Delivery to Client	(99.0, 236.25, 459.0)
F13	Freight Cost	(69.6, 187.20, 377.52)
F14	Delivery to Client	(170.625, 360.0, 617.5)
F14	Freight Cost	(126.0, 286.875, 540.0)
F15	Delivery to Client	(15.0, 63.0, 165.0)
F15	Freight Cost	(39.2, 115.5, 263.9)
F16	Delivery to Client	(86.4, 243.36, 432.0)
F16	Freight Cost	(103.125, 252.0, 467.5)
F17	Delivery to Client	(44.1, 126.0, 282.75)
F17	Freight Cost	(22.5, 81.0, 198.0)
F18	Delivery to Client	(30.0, 102.375, 240.0)
F18	Freight Cost	(19.6, 73.5, 188.5)
F19	Delivery to Client	(18.0, 70.4, 184.8)
F19	Freight Cost	(15.75, 64.0, 171.6)
F20	Delivery to Client	(86.625, 210.0, 413.44)
F20	Freight Cost	(75.0, 204.75, 400.0)
F21	Delivery to Client	(37.8, 113.40, 261.0)
F21	Freight Cost	(15.0, 63.0, 165.0)

For combined rankings across the two objectives (Delivery to Client Date and Freight Cost), Equation 22 is defined to calculate the a total hybrid score:


$$Q_{\mathrm{total},i}=0.5\cdot Q_{\mathrm{Delivery},i}+0.5\cdot Q_{\mathrm{Freight},i}$$ (22)

where $Q_{\mathrm{Delivery},i}$ and $Q_{\mathrm{Freight},i}$ are the hybrid scores for each objective.

## 4 Results

This study harnesses a mix of techniques, including multi-criteria decision-making (MCDM), Data Envelopment Analysis (DEA), and optimization strategies, combining Z-numbers, Failure Mode and Effects Analysis (FMEA), Z-SWARA, Z-WASPAS, and Z-DEA-FMEA methodologies into a hybrid ranking model. Data from ARV and HIV lab shipments, supplemented by expert insights, inform the findings.

Table 13 presents Z-number assessments for Severity (S), Occurrence (O), and Detection (D) across 21 failure modes, with values for Quantity Errors [F14, Delivery S = (7,8,9), O = (6,7,8)], Shipment Mode Selection [F04, Delivery S = (6,7,8)], and Unit Miscalculation [F13, Delivery S = (5, 6.25, 7.5)]. Table 15 lists RPN middle values (e.g., F14 Delivery = 360, F16 Freight = 252). Table 4 provides Z-SWARA weights, and Table 5 reports Z-WASPAS K scores [e.g., F14 Delivery *K* = (0.116, 0.143, 0.189), F16 Freight *K* = (0.141, 0.178, 0.242)]. Table 16 ranks failure modes based on RPN and Z-WASPAS rankings (e.g., F14 Delivery: RPN 1st, Z-WASPAS 1st; F16 Freight: RPN 2nd, Z-WASPAS 1st). Table 14 provides Z-DEA-FMEA efficiencies (e.g., F14 Delivery 𝜃=1.0, F06 Delivery 𝜃=0.7303).

**Table 16:** Comparison of RPN and Z-SWARA-Z-WASPAS rankings for HIV drug supply chain factors by Delivery to Client and Freight Cost objectives.

Factor	RPN delivery TFN	Delivery rank	RPN freight TFN	Freight rank	Z-WASPAS delivery K	Delivery rank	Z-WASPAS freight K	Freight rank
F01	(24.0, 109.44, 264.0)	9	(13.125, 62.4, 169.65)	16	(0.209, 0.293, 0.507)	9	(0.273, 0.376, 0.588)	13
F02	(27.0, 94.5, 222.75)	15	(16.5, 78.4, 204.0)	10	(0.292, 0.413, 0.65)	19	(0.281, 0.405, 0.723)	20
F03	(28.5, 93.6, 224.4)	14	(16.8, 66.15, 174.0)	13	(0.216, 0.299, 0.453)	11	(0.255, 0.356, 0.536)	10
F04	(48.75, 163.2, 342.0)	5	(63.0, 167.875, 375.0)	2	(0.149, 0.192, 0.285)	4	(0.161, 0.209, 0.296)	4
F05	(35.0, 110.0, 250.25)	10	(13.5, 60.48, 167.04)	19	(0.208, 0.285, 0.431)	7	(0.288, 0.417, 0.658)	16
F06	(30.0, 101.25, 240.0)	12	(21.0, 75.6, 191.4)	11	(0.224, 0.313, 0.493)	14	(0.260, 0.359, 0.542)	11
F07	(16.875, 72.0, 185.625)	16	(10.8, 56.0, 156.0)	20	(0.283, 0.411, 0.711)	18	(0.288, 0.416, 0.684)	17
F08	(17.5, 70.0, 178.75)	19	(33.6, 106.05, 243.6)	6	(0.232, 0.322, 0.506)	15	(0.220, 0.302, 0.444)	6
F09	(15.0, 63.0, 165.0)	20	(15.12, 67.2, 180.96)	12	(0.257, 0.366, 0.595)	17	(0.229, 0.321, 0.481)	8
F10	(33.75, 108.0, 247.5)	11	(13.5, 64.0, 178.2)	14	(0.214, 0.296, 0.452)	10	(0.310, 0.446, 0.716)	21
F11	(35.0, 113.75, 260.0)	8	(15.0, 63.0, 165.0)	17	(0.221, 0.309, 0.486)	12	(0.268, 0.374, 0.585)	12
F12	(16.8, 69.3, 182.7)	18	(10.8, 56.0, 156.0)	21	(0.288, 0.419, 0.714)	21	(0.291, 0.421, 0.690)	18
F13	(99.0, 236.25, 459.0)	2	(43.5, 149.76, 343.2)	4	(0.133, 0.171, 0.232)	2	(0.175, 0.226, 0.310)	5
F14	(170.625, 360.0, 617.5)	1	(126.0, 286.875, 540.0)	1	(0.116, 0.143, 0.189)	1	(0.153, 0.194, 0.257)	2
F15	(15.0, 63.0, 165.0)	21	(21.0, 92.4, 245.7)	7	(0.243, 0.339, 0.530)	16	(0.225, 0.305, 0.438)	7
F16	(86.4, 243.36, 432.0)	3	(41.25, 163.8, 357.5)	3	(0.152, 0.188, 0.266)	3	(0.141, 0.178, 0.242)	1
F17	(44.1, 126.0, 282.75)	6	(22.5, 81.0, 198.0)	9	(0.200, 0.275, 0.401)	6	(0.283, 0.386, 0.576)	14
F18	(30.0, 101.25, 240.0)	13	(19.6, 78.75, 208.65)	8	(0.221, 0.312, 0.497)	13	(0.252, 0.347, 0.514)	9
F19	(18.0, 70.4, 184.8)	17	(15.75, 64.0, 171.6)	15	(0.285, 0.414, 0.672)	20	(0.301, 0.427, 0.675)	19
F20	(86.625, 210.0, 414.375)	4	(37.5, 149.5, 340.0)	5	(0.159, 0.204, 0.277)	5	(0.154, 0.197, 0.274)	3
F21	(37.8, 113.4, 261.0)	7	(15.0, 63.0, 165.0)	18	(0.207, 0.286, 0.424)	8	(0.290, 0.401, 0.616)	15

**Figure 2:**
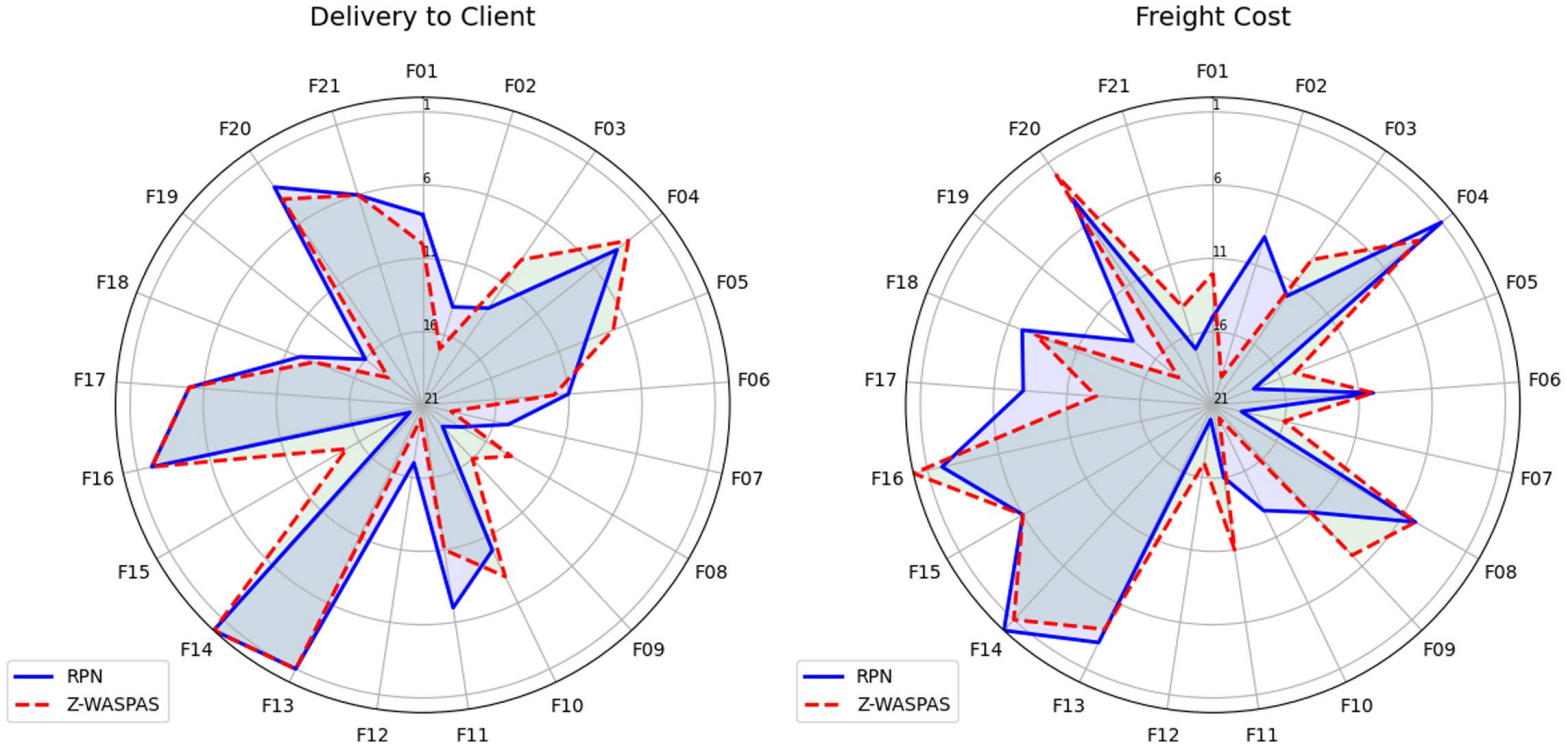
Comparison of RPN and Z-SWARA-Z-WASPAS rankings for HIV drug supply chain.

**Figure 3:**
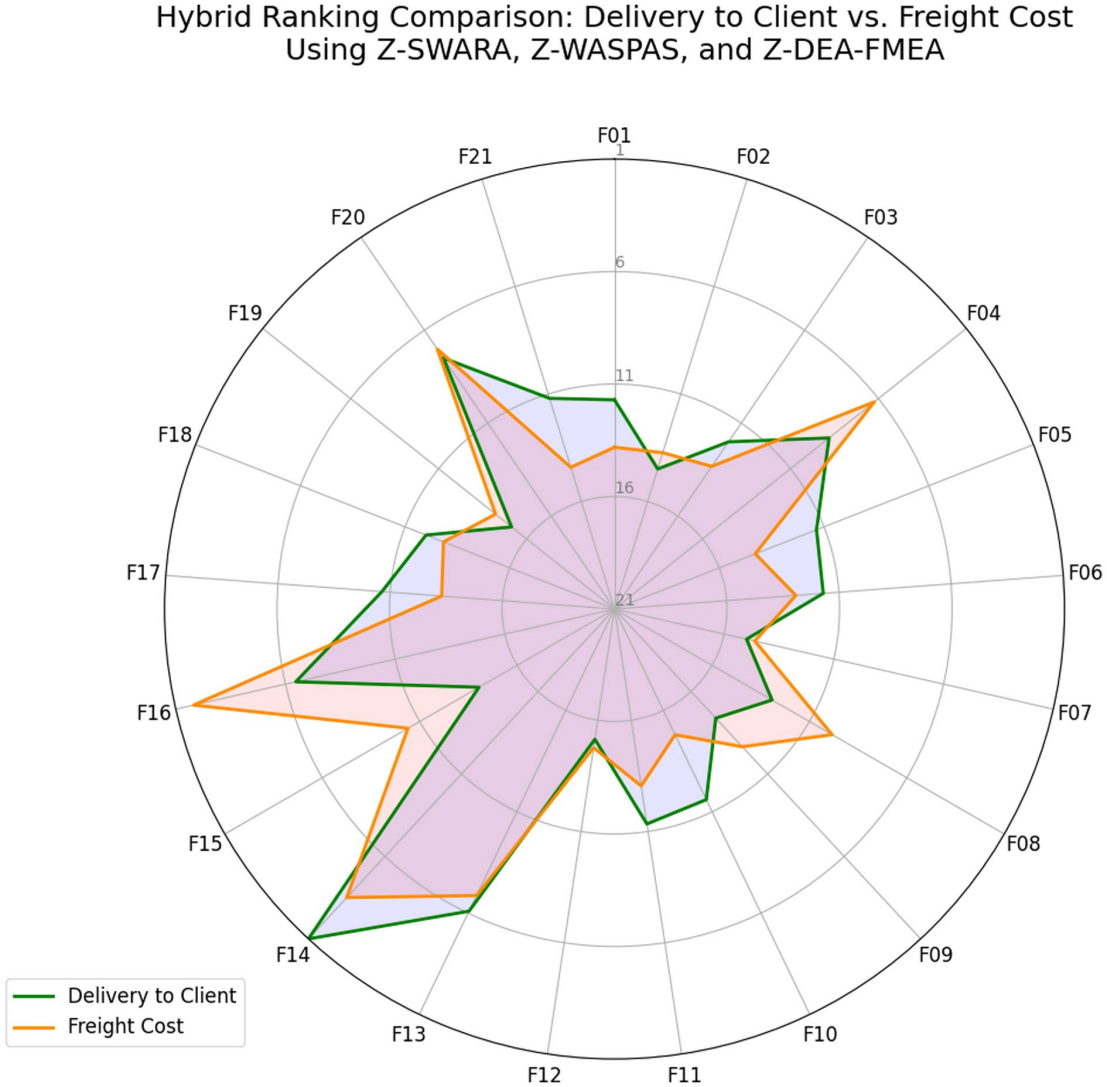
Hybrid rankings based on Delivery to Client date vs. freight cost using Z-SWARA, Z-WASPAS, and Z-DEA-FMEA.

**Figure 4:**
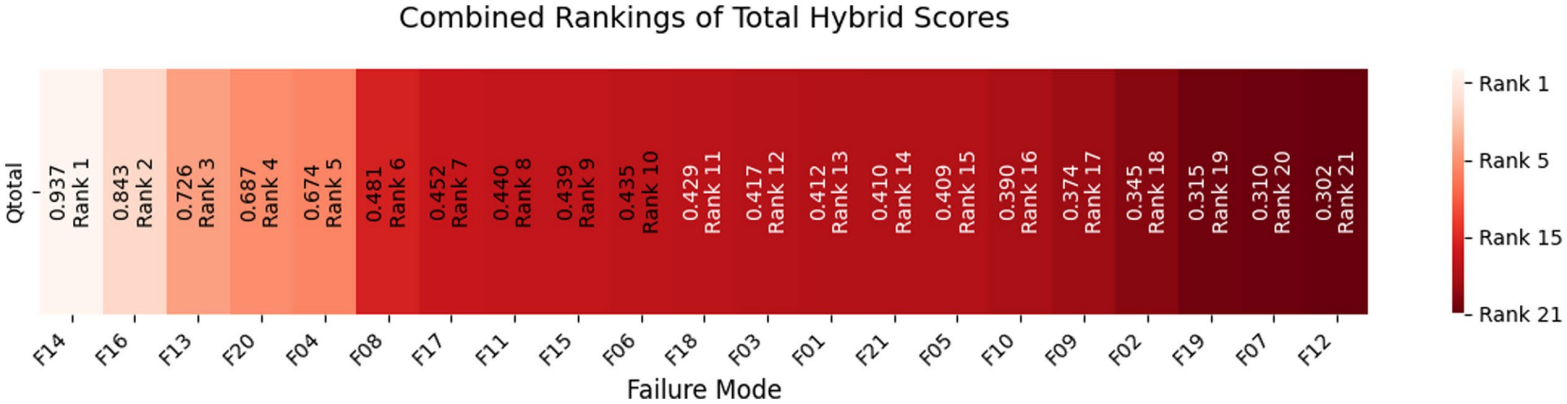
Combined rankings of total hybrid scores for HIV drug supply chain failure modes.

Hybrid rankings (Tables 6, 7) show F14 at 𝑄_𝑖_=1.0 for Delivery and F16 at 𝑄_𝑖_=0.9595 for Freight. Combined rankings (Table 8) list F14 (𝑄_𝑡𝑜𝑡𝑎𝑙_=0.9374), F16 (𝑄_𝑡𝑜𝑡𝑎𝑙_=0.8430), F13 (𝑄_𝑡𝑜𝑡𝑎𝑙_=0.7261), F20 (𝑄_𝑡𝑜𝑡𝑎𝑙_=0.6872), F04 (𝑄_𝑡𝑜𝑡𝑎𝑙_=0.6741), and F06 (𝑄_𝑡𝑜𝑡𝑎𝑙_=0.4347). Figures 2–4 visualize RPN/Z-WASPAS, hybrid, and combined rankings, respectively.

This study provides a detailed analysis of the HIV drug supply chain, identifying critical failure modes and their impact on delivery and cost objectives, setting the stage for targeted improvements. It also provides a robust framework for targeting critical failure modes, enhancing resilience and efficiency.

### 4.1 Analysis of the results of FMEA approach

Failure Mode and Effect Analysis (FMEA) serves as an effective tool for predicting potential system failures and preventing their occurrence. It has also been widely applied in healthcare settings to enhance safety and reliability (75). In this study, focusing on optimizing the HIV drug supply chain, FMEA is employed as the first step to identify risks (76, 77). Twenty-one failure modes that occurred in the ARV and HIV lab supply chain have been identified through a literature review, analysis of the dataset, and finalized approval by experts (78), with their risk factor values determined as Z-numbers, as shown in Table 13. These failure modes, assessed across two components: Delivery to Client Date and Freight Cost (USD) are introduced as follows: “Country Infrastructure” (Country Infrastructure Challenges, F01), “Coordination Failures” (Coordination Failures by Manager, F02), “INCO Term Misalignment” (Vendor INCO Term Misalignment, F03), “Shipment Mode Selection” (Inefficient Shipment Mode Selection, F04), “Delivery Scheduling” (Inaccurate Delivery Scheduling, F05), “Storage Conditions” (Improper Storage Conditions, F06), “Dosage Errors” (Dosage Specification Errors, F07), “Molecule Mismatch” (Molecule/Test Type Mismatch, F08), “Vendor Reliability” (Vendor Reliability Issues, F09), “Brand Delays” (Brand Availability Delays, F10), “Delivery Confirmation” (Delayed Delivery Confirmation, F11), “Product Misclassification” (Product Group Misclassification, F12), “Unit Miscalculation” (Unit of Measure Miscalculation, F13), “Quantity Errors” (Line Item Quantity Errors, F14), “Customs Delays” (Customs Clearance Delays, F15), “Pack Price Discrepancies” (Pack Price Discrepancies, F16), “Demand Forecasting” (Inaccurate Demand Forecasting, F17), “Site Distance Delays” (Manufacturing Site Distance Delays, F18), “First Line Delays” (First Line Designation Delays, F19), “Weight Failures” (Weight-Related Logistical Failures, F20), “Insurance Overruns” (Line Item Insurance Cost Overruns, F21). For brevity, we refer to these failure modes by their short names (e.g., “Country Infrastructure”) throughout this study, with brief designations provided in Table 17. The decision matrix, converted to Z-numbers using Tables 9, 10 (section 3), is presented in Table 13.

**Table 17:** Failure modes addressed by the integrated methodology.

Failure mode	Code
Country Infrastructure	F01
Coordination Failures	F02
INCO Term Misalignment	F03
Shipment Mode Selection	F04
Delivery Scheduling	F05
Storage Conditions	F06
Dosage Errors	F07
Molecule Mismatch	F08
Vendor Reliability	F09
Brand Delays	F10
Delivery Confirmation	F11
Product Misclassification	F12
Unit Miscalculation	F13
Quantity Errors	F14
Customs Delays	F15
Pack Price Discrepancies	F16
Demand Forecasting	F17
Site Distance Delays	F18
First Line Delays	F19
Weight Failures	F20
Insurance Overruns	F21

It is important to note that the HIV drug supply chain is a multifaceted system that is affected by a number of factors. The delivery and distribution of essential medical supplies may be adversely affected by each variable. Understanding and mitigating these risks becomes essential for the efficient and reliable operation of the supply chain. Various variables, ranging from F01 to F21, are assessed using the Failure Modes and Effects Analysis (FMEA) method. In order to assess each variable, three dimensions are considered: severity, occurrence, and detection, rated by experts for both Delivery to Client Date and Freight Cost objectives. In mathematics, Z-numbers represent both deterministic information and uncertainty associated with that information (69). To provide a range of possible values for each variable, two sets of Z-numbers are presented.

#### 4.1.1 Severity

The purpose of this assessment is to determine the potential impact of a failure of the variable. In the event of a failure or compromise of a variable, a higher value (0–10) indicates a greater negative impact.

#### 4.1.2 Occurrence

Using this method, it is possible to determine the likelihood of a variable failure. In general, a higher value indicates a greater likelihood of the variable failing.

#### 4.1.3 Detection

It is a measure of the capability of detecting and correcting failures before they have a negative impact on the outcome. In general, a higher value indicates that the issue is more difficult to detect or correct, while a lower value indicates that monitoring mechanisms are more effective.

The FMEA approach, enhanced by Z-numbers, offers a robust framework for assessing risk factors in the HIV drug supply chain. Table 13 outlines Z-number representations for Severity (S), Occurrence (O), and Detectability (D) across 21 failure modes for Delivery to Client Date and Freight Cost, with expert-validated values, capturing uncertainty.

### 4.2 Risk factor analysis

In this subsection, risk factors will be analyzed based on three dimensions: severity (S), occurrence (O), and detection (D). According to the results obtained from Table 13, values of risk factors for each Failure mode is determined in the form of Z-numbers which reflects uncertainty of information obtained from experts and the reliability of that information as well (69). Risk Priority Numbers (RPNs) are determined based on multiplication of Z-numbers of Severity, Occurrence, and Detection for each Failure Mode, following standard FMEA practices (78) and are presented in Table 15. Decision makers can prioritize failure modes considering RPNs and implement corrective actions based on failure modes with higher RPNs.

For Delivery to Client Date, F14 yields the highest RPN at (170.625, 360.0, 617.5), driven by its high order value and frequent issues, followed by F13 at (99.0, 236.25, 459.0) due to large quantities, and F16 at (86.4, 243.36, 432.0) from volume delays. For Freight Cost, F14 tops with (126.0, 286.875, 540.0), reflecting significant cost impact, closely followed by F16 at (103.125, 252.0, 467.5) and F04 at (63.0, 184.45, 375.0) due to logistics demands. Lower RPNs, like F09 Delivery (15.0, 63.0, 165.0), indicate less critical risks. These baseline RPNs highlight key vulnerabilities, with F14’s dominance across objectives guiding initial prioritization, while subsequent weighted analyses (e.g., Z-WASPAS) refine these rankings.

### 4.3 Z-WASPAS-based risk prioritization

This subsection reports Z-WASPAS-derived risk prioritization results for HIV drug supply chain failure modes across Delivery to Client Date and Freight Costs. Table 4 presents Z-SWARA weights, Table 5 lists Z-WASPAS K scores, and Table 16 provides rankings based on RPN and Z-WASPAS K middle values for 21 failure modes.

4.3.1. Role of Z-SWARA in Z-WASPAS

Here, we harness the Z-SWARA method to weigh the heartbeat of risk assessment, Severity (𝑆), Occurrence (𝑂), and Detection (𝐷) drawing from expert wisdom in the HIV drug supply chain (68, 69). Z-SWARA, an insightful twist on Step-wise Weight Assessment Ratio Analysis, weaves uncertainty and confidence into Triangular Fuzzy Numbers (TFNs) via Z-numbers, setting the stage for Z-WASPAS (Section 4.3.2). Experts crafted through their seasoned insights, employing ranking and fuzzy comparisons grounded in the Z-SWARA framework. Table 4 presents the resulting weights for each objective.

Table 4 reports Z-SWARA weights for Severity (S), Occurrence (O), and Detection (D) across Delivery to Client Date and Freight Costs, derived from expert assessments. For Delivery to Client Date, the weights are 𝑤_𝑆=_(0.47, 0.54, 0.62), 𝑤_𝑂_=(0.33, 0.36, 0.39), and 𝑤_𝐷_=(0.14, 0.17, 0.21), with middle values of 0.54, 0.36, and 0.17, respectively, indicating Severity’s higher priority. For Freight Cost, the weights are 𝑤_𝑆_=(0.42, 0.48, 0.54), 𝑤_O_ = (0.32, 0.35, 0.39), and 𝑤_𝐷_=(0.15, 0.18, 0.20), with middle values of 0.48, 0.35, and 0.18. These weights inform Z-WASPAS K scores in Table 5.

#### 4.3.1 Z-WASPAS rankings

The Z-WASPAS method is applied to rank failure modes (F01–F21) in the HIV drug supply chain, using the Z-SWARA weights from Table 4 and Z-numbers from Table 13. Z-WASPAS combines the Weighted Sum Model (WSM) and Weighted Product Model (WPM) to produce a combined score (K) for each failure mode, as detailed in section 3.5. Table 5 presents the WSM, WPM, and K scores as triangular fuzzy numbers (TFNs) for both objectives: Delivery to Client Date and Freight Cost.

The Z-WASPAS methodology is distinctive in its strong dependence on expert judgments. This table comes from experts’ opinions, covering factors F01 to F21. Examples include F14 Delivery *K* = (0.116, 0.143, 0.189), F16 Freight *K* = (0.141, 0.178, 0.242), F13 Delivery *K* = (0.133, 0.171, 0.232), and F20 Freight *K* = (0.154, 0.197, 0.274).

### 4.4 Comparative analysis and hybrid prioritization of risk factors

This section compares the risk prioritization methods applied in the HIV drug supply chain RPN and Z-WASPAS and integrates them with Z-DEA-FMEA to develop a hybrid ranking of failure modes across Delivery to Client Date and Freight Costs. The comparative analysis highlights the strengths and limitations of each method, while the hybrid approach leverages their combined insights to enhance decision-making. Table 16 compares RPN and Z-WASPAS rankings, Table 14 presents Z-DEA-FMEA efficiencies, and Tables 6–8 provide hybrid and combined rankings for 21 failure modes.

#### 4.4.1 RPN and Z-WASPAS rankings

Table 16 reports RPN rankings (Table 15) and Z-WASPAS rankings (Table 5) for 21 HIV drug supply chain failure modes across Delivery to Client Date and Freight Costs, as defined in Table 17.

For Delivery, top ranks are F14 (RPN middle = 360.0, Z-WASPAS K middle = 0.143, 1st), F13 (236.25, 0.171, 2nd), F16 (243.36, 0.188, 3rd), F04 (163.2, 0.192, 4th), and F20 (210.0, 0.204, 5th). For Freight, top ranks are F16 (252.0, 0.178, 1st), F14 (286.875, 0.194, 2nd), F20 (149.5, 0.197, 3rd), F04 (184.45, 0.209, 4th), and F13 (187.2, 0.226, 5th). Failure mode F06 ranks 12th (101.25, 0.313) for Delivery and 11th (75.6, 0.359) for Freight. Rank differences across objectives include F16 (Delivery: RPN 3rd, Z-WASPAS 3rd; Freight: RPN 2nd, Z-WASPAS 1st) and F04 (Delivery: RPN 5th, Z-WASPAS 4th; Freight: RPN 2nd, Z-WASPAS 4th). Figure 2 visualizes these rankings.

#### 4.4.2 Z-DEA-FMEA efficiency analysis

Sustainable healthcare projects, such as the HIV drug supply chain, demand robust evaluation methods to prioritize risks effectively. The Z-DEA-FMEA technique (section 3.6) addresses this by using Z-numbers to manage uncertainty in expert opinions, leveraging data from Table 13. It assesses how efficiently each failure mode converts risk inputs (Severity, Occurrence) into impact (output) across Delivery to Client Date and Freight Cost objectives, complementing the RPN and Z-WASPAS rankings in section 4.4.1.

Table 12 reports Z-DEA-FMEA inputs (Severity 𝑆_𝑎𝑣𝑔_, Occurrence 𝑂_𝑎𝑣𝑔_) from Table 13 and outputs (reciprocal Z-WASPAS 1/𝐾_𝑎𝑣𝑔_) from Table 5 for 21 HIV drug supply chain failure modes across Delivery to Client Date and Freight Costs. Examples include F14 (Delivery: 𝑆_𝑎𝑣𝑔_=8.097, 𝑂_𝑎𝑣𝑔_=7.067, 1/𝐾_𝑎𝑣𝑔_=6.696; Freight: 7.083, 5.875, 4.967), F20 (Delivery: 5.25, 4.8, 4.688; Freight: 8.097, 5.417, 4.8), and F06 (Delivery: 3.6, 2.1, 2.913; Freight: 2.8, 3.375, 2.584).

Table 14 lists the final efficiencies (𝜃) for 42 modes, with top values for F14 (Delivery: 1.0; Freight: 0.6838), F16 (Delivery: 0.6666; Freight: 1.0), F20 (Delivery: 0.6636; Freight: 0.6471), F04 (Delivery: 0.5536; Freight: 0.6881), and F06 (Delivery: 0.7303; Freight: 0.6242). F01 scores 0.6341 (Delivery) and 0.5998 (Freight).

4.4.3 Hybrid Rankings Using Z-SWARA, Z-WASPAS, and Z-DEA-FMEA integration.

To enhance the prioritization of failure modes within the HIV drug supply chain, we integrated Z-DEA-FMEA with Z-SWARA and Z-WASPAS, combining RPN (Table 15), Z-WASPAS scores with Z-SWARA weights (Tables 4, 5), and Z-DEA efficiencies (Table 14). For each failure mode, we compute a hybrid score $Q_{i}$ as described in section 3:


$$\begin{array}{c} \begin{array}{c} Q_{ij}=\frac{1}{3}\cdot \mathrm{Norm}.{\mathrm{RPN}}_{i}+\frac{1}{3}\cdot \mathrm{Norm}.K_{ij}+\frac{1}{3}\cdot \mathrm{Norm}.\theta_{j} \end{array} \end{array}$$

Tables 6, 7 report hybrid rankings (𝑄_𝑖_) for 21 HIV drug supply chain failure modes across Delivery to Client Date and Freight Costs, combining RPN (Table 15), Z-WASPAS scores (Table 5), and Z-DEA-FMEA efficiencies (Table 14), ranging from 1.0 (F14 Delivery) to 0.2927 (F19 Delivery) and 0.9595 (F16 Freight) to 0.3101 (F10 Freight). Table 8 presents combined rankings (𝑄_𝑡𝑜𝑡𝑎𝑙_), averaging 𝑄_𝑖_ from both objectives, spanning 0.9374 (F14) to 0.3022 (F12). For Delivery (Table 6), ranks are F14 (𝑄_𝑖_=1.0, 1st), F13 (0.7454, 2nd), F16 (0.7265, 3rd), F20 (0.6753, 4th), F04 (0.6098, 5th), F11 (0.4830, 8th), and F08 (0.4042, 15th). For Freight (Table 7), ranks are F16 (0.9595, 1st), F14 (0.8747, 2nd), F20 (0.6991, 3rd), F04 (0.7385, 4th), F13 (0.7067, 5th), and F08 (0.5585, 6th). Table 8 lists combined ranks: F14 (0.9374, 1st), F16 (0.8430, 2nd), F13 (0.7261, 3rd), F20 (0.6872, 4th), F04 (0.6741, 5th), F08 (0.4813, 6th), and F15 (0.4388, 9th).

Table 6 shows Delivery rankings, with 𝑄_𝑖_ scores ranging from 1.0 (F14) to 0.2927 (F19), where F14, F13, and F16 lead, while F08 shifts from 13th in Z-WASPAS (Table 16) to 15th and F11 from 12th to 8th. Figure 3 visualizes these 𝑄_𝑖_ scores.

Table 7 lists Freight rankings, with 𝑄_𝑖_ scores spanning 0.9595 (F16) to 0.3101 (F10), highlighting F16, F14, and F20 as top risks, and F08 rising from 15th in Z-WASPAS (Table 16) to 6th. Figure 3 illustrates these 𝑄_𝑖_ rankings.

Table 8 reports combined rankings, with 𝑄_𝑡𝑜𝑡𝑎𝑙_ ranging from 0.9374 (F14) to 0.3022 (F12), where F14 and F16 dominate, F08 shifts from 15th in Z-WASPAS Delivery (Table 16) to 6th, and F15 reaches 9th. Figure 4 visualizes these 𝑄_𝑡𝑜𝑡𝑎𝑙_ scores.

This hybrid methodology, leveraging Z-numbers to address uncertainty, enhances the prioritization of failure modes in complex supply chains. The rankings in Tables 6–8 and visualized in Figure 4, provide a robust foundation for targeted interventions, optimizing both logistics and cost efficiency in the HIV drug supply chain.

## 5 Discussion

To ensure the distribution of antiretrovirals (ARVs) to millions globally, it is crucial to have effective HIV drug supply chains. The hybrid framework employed in this research tackles uncertainty in a manner that surpasses traditional Failure Mode and Effects Analysis (FMEA) by integrating Z-SWARA, Z-WASPAS, and Z-DEA-FMEA with Z-numbers. The primary risks identified concerning delivery timelines and freight costs include Quantity Errors (F14), Pack Price Discrepancies (F16), and Unit Miscalculation (F13), as detailed in Tables 6–8, 14, 16. This section evaluates the results from each method, elucidate their mathematical and practical significance, and highlight any limitations. This analysis offers a comprehensive view by linking data to tangible improvements in logistics.

### 5.1 Contribution of Z-SWARA in risk prioritization

Utilizing the expert weights outlined in Table 4, Z-SWARA assigns priority to risks, favoring severity with Delivery rated at 0.54 and Freight at 0.48. Consequently, F14 ranks first with a total 𝑄_total_ of 0.9374 (Table 8), and its significant 𝑆_𝑎𝑣𝑔_ of 8.097 (Table 12) reflects stockouts that lead to delays in clinic treatments. The focus on Occurrence, particularly with Freight rated at 0.35, benefits F04, which ranks fifth with a 𝑄_total_ of 0.6741, highlighting real transportation issues such as customs delays. While Z-SWARA’s triangular fuzzy number (TFN) weights enhance the raw scores of the Risk Priority Number (RPN), there is a risk that expert bias could skew the results if they are not cross-validated.

### 5.2 Z-WASPAS and uncertainty insights

Z-WASPAS occupies Triangular Fuzzy Numbers (TFNs) to quantify uncertainty, as Table 16 shows F14’s Delivery K (middle 0.143, 1st) indicates a high frequency of quantity discrepancies, with procurement errors negatively impacting the flow of ARVs in practice, in addition F16’s Freight K (0.178, 1st) exceeds F14 (0.194, 2nd) and captures the financial impact of price volatility, which is a major concern for suppliers in the global health sector. The range of TFNs (e.g., F16: 0.141–0.242) offers a mathematical framework for risk assessment, which is more thorough than the single-point method of the Risk Priority Number (RPN) in Table 15, and F20’s Freight K (0.197, 3rd) is linked to overweight shipments, which is a significant cost factor. Expert input shapes this precision, though context specificity may limit its reach.

### 5.3 Z-DEA-FMEA’S efficiency angle

Z-DEA-FMEA adds an efficiency angle, balancing risk with operational performance. This balance between risk and efficiency ensures that the hybrid framework provides actionable insights for optimizing resource use in the HIV drug supply chain. Table 14 measures risk-to-impact efficiency, revealing F14’s dominance (𝜃=1.0 Delivery, 0.6838 Freight) and F16’s Freight lead (𝜃=1.0). F06’s rise to 10th (𝑄_𝑡𝑜𝑡𝑎𝑙_=0.4347, Table 8) from 14th in Z-WASPAS (*K* = 0.313, Table 16) with 𝜃=0.7303 (Delivery) shows tracking efficiency, a practical fix for rural delays. F20’s Freight 𝜃=0.6471 (3rd, Table 7) flags weight costs, common in bulk shipping. Mathematically, 𝜃 balances inputs (Table 12, e.g., F14 𝑆_𝑎𝑣𝑔_ = 8.097) against 1/𝐾_𝑎𝑣𝑔_, unlike RPN’s risk-only lens, though it skips patient-level effects. Building on this, integrate ML-based optimization as in (79) to dynamically predict and resolve prioritized risks under constraints.

### 5.4 Method comparisons and rank shifts

This study outstrips traditional FMEA (75, 80), which lacks efficiency metrics, and standalone MCDM studies, often weak on uncertainty. Unlike studies (41), who cut vaccine costs via delivery redesign without risk ranking, our framework merges both, echoing sustainability focus with added Z-number precision (59, 60).

Table 16 presents a comparison between RPN and Z-WASPAS rankings, highlighting F14’s consistent first position in Delivery (RPN = 360.0, *K* = 0.143). This indicates that quantity errors significantly impact stock levels, posing a genuine challenge for remote clinics and healthcare facilities. Meanwhile, F16’s Freight ranking shifts from second place in RPN (252.0) to first in Z-WASPAS (*K* = 0.178), underscoring the financial implications of pricing, influenced by the Severity weight (Table 4, 𝑤_𝑆_=0.48) which differs from the straightforward RPN count. In Table 14, the Z-DEA-FMEA efficiencies redirect attention, with F06 advancing to 10th place overall (𝑄_𝑡𝑜𝑡𝑎𝑙_=0.4347, Table 8) from its previous 14th position in Z-WASPAS (*K* = 0.313). Its 𝜃 =0.7303 (Delivery) enhances efficiency compared to RPN’s risk-focused perspective (101.25). Additionally, F08 rises to 6th place in the Hybrid (𝑄_𝑡𝑜𝑡𝑎𝑙_=0.4813) from 13th in Z-WASPAS Delivery (*K* = 0.322), with its Freight 𝜃=0.7757 addressing clinical cost risks, molecule mismatches can delay treatment regimens, presenting a practical obstacle.

Tables 6, 7 refine this, F14’s Delivery 𝑄𝑖=1.0 and Freight 𝑄_𝑖_=0.8747 dominate, but F20’s Freight rise to 3rd (0.6991) from RPN 5th (149.5) highlights weight’s cost impact (Table 14, 𝜃=0.6471), a shipping burden in bulk ARV transport. The logistics risks have been mitigated by a reduction in F04’s freight, which has dropped from RPN 2nd (184.45) to 4th (0.7385) reflecting enhanced efficiency (𝜃 = 0.6881). Combined 𝑄𝑡𝑜𝑡𝑎𝑙 presented in Table 8 indicate that F14 (0.9374) and F16 (0.8430) represent the highest risks, while F13 (0.7261, 3rd) remains consistent, with ongoing unit errors noted in inventory records. The observed low efficiency across objectives (Table 14, 𝜃=0.6739, 0.6525) and K scores (0.419, 0.411) are evident in the lower rankings of F12 (0.3022, 21st) and F07 (0.3104, 20th), which reflect minor operational issues that are often overshadowed by procurement and logistical deficiencies.

From a mathematical perspective, the TFN range of Z-WASPAS (for instance, F16 Freight: 0.141–0.242) accounts for the uncertainties associated with RPN omissions, whereas Z-DEA-FMEA’s 𝜃 (for example, F08 Freight = 0.7757) introduces an efficiency dimension that is lacking in the straightforward calculations of RPN. Real-world analogies can be drawn, F14 corresponds to stock market crises, while F16 relates to budgetary pressures. However, variations such as F15 (9th, 𝑄_𝑡𝑜𝑡𝑎𝑙_ = 0.4388) from Z-WASPAS’s 16th (K = 0.339 Delivery) highlight the often-overlooked costs associated with customs, representing a subtle yet significant challenge in logistics.

### 5.5 Limitations

While the hybrid framework offers significant insights, it also presents certain limitations that warrant further investigation. Though we have boosted the method with Z-numbers, the research still leans on expert opinions for the Z-SWARA and Z-WASPAS techniques, and that might result in an element of subjectivity. Moreover, our data is limited to the HIV supply chain, so additional validation might be needed to make sure that the results are transferable to other scenarios, such as vaccine distributions. It is worth mentioning that, the framework tends to overlook downstream health outcomes, such as how the timely delivery of antiretroviral (ARV) medications influences patient adherence and viral suppression rates, focusing instead on supply chain efficiency metrics like delivery timelines and transportation costs.

## 6 Conclusion

This study advances HIV drug supply chain management by integrating Z-SWARA, Z-WASPAS, and Z-DEA-FMEA into a hybrid framework that outstrips traditional FMEA. Table 8 ranks Quantity Errors (F14, 𝑄_𝑡𝑜𝑡𝑎𝑙_=0.9374), Pack Price Discrepancies (F16, 0.8430), Unit Miscalculation (F13, 0.7261), Weight Failures (F20, 0.6872), and Shipment Mode Selection (F04, 0.6741) as top risks across delivery timelines and freight costs balancing substantivity and resilience as outlined in Section 1.

Z-numbers enhance prioritization over conventional methods, revealing critical stock shortages (F14) and pricing volatility (F16), often missed by RPN alone. Observations regarding efficiency, illustrated by the increase in F06 (𝑄_𝑡𝑜𝑡𝑎𝑙_ = 0.4347, 10th) with 𝜃=0.7303, show how monitoring and tracking can reduce delays. These insights enable managers to focus on significant inefficiencies, optimizing operations in areas most affected by disruptions. Furthermore, this research tackles a critical issue: each delay or budget overrun adversely impacts lives in resource-limited regions. Beyond prior studies, by pinpointing actionable priorities, this framework serves as a valuable resource to enhance access to ARVs, reduce expenses, and maximize limited resources, effectively addressing gaps in the global battle against HIV/AIDS with accuracy and intent.

### 6.1 Future work

Future study might improve this hybrid framework by using Neural Networks to stabilize price (F16, 2nd), Hybrid Salp Swarm approaches to optimize shipment schedules (F04, 5th), or Genetic Algorithms to increase quantity accuracy (F14, _𝑄𝑡𝑜𝑡𝑎𝑙_ = 0.9374). While machine learning might modify logistics in response to regional bottlenecks, such the infrastructure concerns noted in F01, real-time analytics could be utilized to forecast unit delays (F13, 3rd). Assessing this method within the context of vaccine or emergency drug supply chains would determine its applicability beyond HIV logistics. Moreover, exploring the connections between key risks, like the delays highlighted in F14 and patient results, such as viral suppression levels, could tie supply chain performance directly to better health outcomes. Such steps aim to evolve the framework into a versatile, expandable tool that addresses bottlenecks and guarantees the prompt arrival of critical medications.

## Data Availability

The original contributions presented in the study are included in the article/supplementary material, further inquiries can be directed to the corresponding author.
